# Competitive Binding of UBA52 and HOPX Modulates β-catenin Stability in Colorectal Cancer in the Context of High-Iron Intake

**DOI:** 10.7150/ijbs.126038

**Published:** 2026-04-16

**Authors:** Xiangjun Liu, Tong Tang, Xi Wang, Jianhua Feng, XiangJie Yang, Yajun Luo, Yujun Huang, Lu Xu, Banghui Liu, Nan Wang, Yikun Luo, Hefei Tian, Shubin Wang, Lingxiao Huang, Zhenni Xu, Hai Hu, Chao Liu, Xudan Lei, Jinyi Lang, Dengqun Liu

**Affiliations:** 1 Precision Radiation in Oncology Key Laboratory of Sichuan Province, Sichuan Cancer Hospital & Institute, Sichuan Clinical Research Center for Cancer, Sichuan Cancer Center, School of Medicine, University of Electronic Science and Technology of China, Chengdu 610041, China.; 2 Department of Experimental Research, Sichuan Cancer Hospital & Institute, Sichuan Provincial Engineering Research Center for Tumor Organoids and Clinical Transformation, Sichuan Clinical Research Center for Cancer, Sichuan Cancer Center, School of Medicine, University of Electronic Science and Technology of China, Chengdu 610041, China.; 3 Department of Colorectal Surgery, Sichuan Cancer Hospital & Institute, Sichuan Provincial Engineering Research Center for Tumor Organoids and Clinical Transformation, Sichuan Clinical Research Center for Cancer, Sichuan Cancer Center, School of Medicine, University of Electronic Science and Technology of China, Chengdu 610041, China.

**Keywords:** iron diet, Hopx^+^ ISCs, colorectal cancer, Wnt/β-catenin pathway, UBA52

## Abstract

Over-intake of dietary iron and hereditary iron overload are implicated in colorectal cancer (CRC) carcinogenesis, yet the mechanistic basis of how iron-mediated signaling leads to oncogenesis remains enigmatic. Here we demonstrate that high iron diet augments the regenerative capacity of Hopx^+^ intestinal stem cells (ISCs) rather than Lgr5^+^ ISCs to functionally contribute to colon tumor formation. Mechanistically, high iron activates a robust Wnt/β-catenin signaling in ISCs to enhance the proliferation of colonic cells in a Hopx-dependent manner. Furthermore, Wnt/β-catenin induction is attributed to Hopx stabilizing β-catenin protein by directly inhibiting the interaction of β-catenin with UBA52, which targets ubiquitination degradation of β-catenin. This study thus identifies an iron-triggered pathway regulating intestinal tumorigenesis and indicates that iron interventions may complement current prevention and treatment strategies for CRC, and Hopx is a previously unrecognized regulator of β-catenin and a therapeutic target of CRC.

## Introduction

Whether dietary patterns can enhance tissue regeneration without promoting cancer initials is a key question in regenerative medicine. Hereditary iron overload and over-consumption of iron-rich diets are relevant to growth-promoting signaling networks. For example, a significant link between dietary iron intake and increased incidence in several cancers including CRC was demonstrated in several epidemiological studies 1-3. Moreover, overload-iron in collusion with genetic factors reactivates telomerase to promote colorectal tumor initiation 4. However, the mechanism of how iron-mediated role contributes to stem-cell organismal benefits and gives their importance in tumorigenesis remains enigmatic. Several findings have revealed that iron drives corresponding epigenetic and metabolic networks, remaining stemness conferring cell-fate control in adult stem cells 5-7. Previous studies have reported an enhanced tumor-growth effect mediated by iron was conversely blocked by iron chelators 8, 9. However, it is unclear whether or how high iron levels may contribute to the initiation and progression of CRC. Furthermore, whether it might be a useful strategy for CRC treatment or prevention through targeting intratumoral iron remains enigmatic. Here we aimed to dissect the effects of high-iron diet on stem cell-mediated regeneration and tumor initiation in the mammalian intestine.

The rapidly renewing mammalian intestinal epithelium responds to diet-induced physiological cues to dynamically alter intestinal composition 10, 11. Intestinal stem cells (ISCs) which located at the base of intestinal crypts dominantly maintain the intestinal epithelial lining, which is a precursor to digestion and absorption 10, 12. The turnover of intestinal epithelium in tissue homeostasis is mediated by two distinct populations of intestinal stem cells: highly proliferative crypt-base cells marked by Lgr5 12 and slow-cycling stem cells marked by Hopx, Bmi1, Lrig1, Tert 13-16. The activity of the ISCs is dominated by growth-factors derived from niche and development-regulating networks, such as WNT, NOTCH and BMP, but are also influenced by metabolites and diets 17-20.

Intestinal dynamics may be controlled by host diet and nutrients through direct and indirect effects of ISCs 19, 21, 22. Here we demonstrate that high-iron increases robust Wnt signaling pathway in ISCs, which give rise to intestinal early dysplasias to exert tumor-promoting role in human intestine 15, 23. Thus, diverse cues capable of intestinal adaptation are integrated by ISCs and their niches, but further investigation is required to explain how nutritional or metabolic events orchestrate ISCs behavior and CRC formation. Recent studies of protein regulation in regulating the stemness and carcinogenicity of other tissues attracted us to study how ISCs orchestrate the high-iron response in CRC 24, 25. Here we demonstrated that the high-iron diet promotes ISC-mediated regeneration of colon tumorigenicity by upregulating β-catenin in Hopx^+^ stem cell, and Hopx competes with β-catenin for binding to UBA52, thereby inhibiting UBA52-mediated polyubiquitination of β-catenin and its subsequent proteasomal degradation.

## Materials and Methods

### Animal studies

Hopx-CreERT2, Lgr5-eGFP-IRES-CreERT2, Rosa-LSL-tdTomato, and iDTR mice were purchased from Jackson Laboratory. Lgr5-IRES-CreERT2 mice were crossed with Rosa26LSL-tdTomato mice, while Hopx-CreERT2 mice were crossed with Rosa-LSL-tdTomato or iDTR mice to generate HopxCreERT2; RosatdTomato and HopxCreERT2;iDTR mice, respectively. C57BL/6 and NOD.Cg-Prkdcscid IL2rgtm1Wjl/SzJ (NSG) mice were obtained from GemPharmatech (Nanjing, China). All mice were housed in an SPF facility with a 12h/12h light-dark cycle and adjusted humidity, with free access to standard lab food and water. Male and female mice aged 8-12 weeks were used in the study. The experimental sample size was determined by experimental needs and published literature without predetermination. Both genders were used for AOM/DSS-induced rectal cancer and organoid generation. In NSG mouse subcutaneous transplantation experiments, investigators monitored tumor size regularly and randomly assigned male and female mice to the experimental group. All procedures complied with the Guidelines for the Care and Use of Laboratory Animals and were approved by the Ethics Committee of Sichuan Cancer Hospital & Institute (SCCHEC-04-2023-017).

### Diets

Iron-diet was designed according to previous studies 1, 36. Before the start of any experimental interventions, mice received either a standard mature rodent diet (normal iron content: 45 ppm) or diets with extra iron supplementation, including medium-iron (100 ppm) and high-iron (500 ppm) formulations, for no less than 7 days. These iron-containing diets were continuously administered to the mice throughout the entire research period; however, the iron diet pretreatment was halted at the experimental terminal time point following 7 days of initial administration.

### AOM/DSS induced colon tumorigenesis

For orthotopic colon cancer model induction, mice were intraperitoneally injected with 10 mg/kg body weight of the mutagen AOM (A5486, Sigma-Aldrich). Twenty-four hours later, they received drinking water containing 2% DSS (#216011080, MP Biomedicals) continuously for 6 days, followed by regular drinking water for 12 days; this DSS administration cycle was repeated twice more. Stool blood and mouse weight changes were monitored regularly to evaluate tumor progression. Both genders were used for AOM/DSS-induced rectal cancer and organoid generation.

### Lineage tracing and cell ablation

To ensure single-cell clonal recombination in tumors, HopxCreERT2/RosaTd and Lgr5-eGFP-IRES-CreERT2 CRC mice received an intraperitoneal injection of diluted tamoxifen (T5648, Sigma) at 10 mg/kg body weight 24 h before the scheduled experiment. Following tamoxifen induction, experiments were completed, mice were sacrificed, and samples were fixed. For ablation during injury, HopxCreER/RosaDTR CRC mice and their WT littermate controls were treated with DSS for 6 days, followed by a 12-day water washout period without DSS. Starting from each DSS withdrawal, three total doses of tamoxifen and diphtheria toxin (DT, 50 μg/kg, Sigma) were administered, and mice were sacrificed after the final water washout.

### Tumor organoid culture

For organoid culture, fresh surgical specimens of colorectal cancer (from patients or mice) were cut into 0.5 cm fragments, followed by enzymatic dissociation. The digestion medium consisted of RPMI supplemented with 10% FBS and 1% Primocin, containing 1 mg/ml collagenase I, 50 μg/ml DNase, and 0.5 mg/ml Dispase (all from Sigma-Aldrich), and the dissociation was performed for 30-40 min. The resulting supernatant was washed and filtered through a 70 μM cell strainer. Isolated cells were embedded in Matrigel (Corning) mixed with Advanced DMEM/F-12 Medium (Gibco), supplemented with GlutaMax, HEPES, Pen/Strep, N2 Supplement, B27 Supplement (all from Invitrogen), and n-acetylcysteine (Sigma-Aldrich). Niche factors including EGF, R-Spondin1, and Noggin (Invitrogen) were added to the medium. For AK organoid culture, 200 μg/ml Geneticin and 2 μg/ml puromycin were added. To improve organoid survival, 10 μM ROCK inhibitor Y27632 was added during the first three days of culture initiation. Organoids were passaged every 7-10 days based on their size and density. The culture medium was discarded, and organoids were released from Matrigel using ice-cold Cell Recovery Solution (Corning, 354253) for 10-20 min. Released organoids were centrifuged at 60 g for 5 min, then further dissociated with TrypLE Express (Invitrogen) at 37 °C for 5 min to obtain small cell clusters. These clusters were reseeded in Matrigel, and the medium was refreshed every 2-3 days. For long-term preservation, organoids or dissociated cells were resuspended in cold Recovery Cell Culture Freezing Medium and stored at -80 °C or in liquid nitrogen.

### Genome editing of PDO

For the generation of human naive AK colorectal cancer (CRC) organoids, APC^min/+^ and KRAS^G12D^ lentiviruses were obtained from Genechem (Shanghai, China). Briefly, human CRC organoids were dissociated into single cells with TrypLE (Invitrogen), and 1×10^5^ cells were resuspended in DMEM/F12 medium supplemented with 5 μg/ml polybrene (infection reagent) and lentivirus (MOI = 20). The cell suspension was incubated at 37 °C in a CO2 incubator for 6 h, after which the organoids were embedded in 50 μl Matrigel and cultured in 500 μl medium. At 48h post-gRNAs transduction, 2 μg/ml puromycin and 200 μg/ml Hygromycin were added to the medium for positive cell screening to maintain AK organoid culture. To prepare human AK-HOPX^-ko^ organoids, HOPX knockout lentivirus was purchased from Genechem (Shanghai, China), with the guide RNA (sgRNA) sequence for HOPX as follows: GTCCTCTGTGGGGCCGCTCG. AK organoids were transfected with HOPX^-KO^ lentivirus following the same protocol described above.

### Xenograft experiments

Human AK-HOPX^-wt^ and AK-HOPX^-ko^ organoids were incubated with Cell Recovery Solution (Corning) to detach them from Matrigel, followed by dissociation into small fragments or uniform single cells using TrypLE Express. A total of 1×10⁶ dissociated organoid cells were resuspended in 100 μl of a 1:1 mixture of PBS and Matrigel, which was then subcutaneously injected into the flanks of NSG mice. When tumor volume reached ~100 mm³ (calculated by the formula: ((length × width²)/2), mice were fed an iron-replete diet (500 ppm). Tumor dimensions were measured every 3 days until euthanasia. All animal experiments complied with the protocol approved by the Ethical Committee of Sichuan Cancer Hospital (SCCHEC-04-2023-017).

### Human derived research material

Following surgical resection, a portion of the patient-derived tumor tissues was fixed in 4% paraformaldehyde to meet the requirements of subsequent experimental procedures. Prior to this study, human tumor organoids had been successfully constructed using tissues obtained from colorectal adenocarcinoma patients. All the experimental procedures described were reviewed and approved by the Ethics Committee of Sichuan Cancer Hospital, with the approval number SCCHEC-02-2024-069.

### Quantitative RT-PCR

Total RNA was extracted using RNAiso Plus (9109, TaKaRa, Japan) and detected by a NanoDrop2000 spectrophotometer (Thermo Scientific) following the manufacturer's recommendations. Hifair II 1st Strand cDNA Synthesis SuperMix (11137ES60, YEASEN) was used to synthesize cDNA, and qPCR was performed using Hieff qPCR SYBR Green Master Mix (11203ES08, YEASEN). Expression data were normalized to β-actin mRNA levels. Primer sequences were listed in the Supplementary Table 1.

### Organoid transfection

The transfection mixture (comprising 250 μl Opti-MEM, 10 μl Lipofectamine 2000, and either 10 μg plasmid or 25 μM siRNA) was prepared in advance and allowed to stand for 20 minutes. Organoid cells were incubated with the transfection mixture for 24 hours, after which the culture medium was substituted with regular medium. RNA samples were collected 48 hours later to validate the efficiency of gene overexpression and knockdown. Both the plasmid and siRNA were acquired from HANBIO (Shanghai, China), and the sequences of the siRNAs utilized in this study are presented in Supplementary Tables 2.

### Immunostaining

Immunostaining was performed as previously described 26. Briefly, tissues and organoids were fixed with 4% paraformaldehyde. 6 μm OCT frozen tissue sections or 5 μm paraffin-embedded tissue sections were processed for immunostaining using a standard histological protocol. The following primary antibodies were used: Hopx (HPA030180, Sigma-Aldrich, 1:500), Ki67 (ab1667, Abcam, 1:200), β-catenin (610153, BD Biosciences, 1:200). Sections were then incubated with the corresponding secondary antibodies. DAB kit (ZSBio, Beijing, China) was used for color development in immunohistochemistry, nuclei were stained with hematoxylin (blue), and images were captured using an Olympus BX53 microscope for visualization.

For immunofluorescence staining after primary antibody incubation, sections were incubated with fluorochrome-labelled specific secondary antibodies and mounted with ProLong^TM^ Gold Antifade Mountant with DAPI (Invitrogen). Fluorescent images were acquired with ZEISS Axio Observer with Apotome 3 and analyzed using ImageJ.

### Perl's iron staining

Iron staining of the tissue samples was performed utilizing the Prussian Blue Stain Kit (ab150674, Abcam). In brief, slides bearing the tissue sections were incubated in iron staining solution for a 5-minute period, followed by rinsing with phosphate-buffered saline and distilled water successively. Subsequently, the tissue sections were subjected to nuclear fast red counterstaining for 5 minutes, and the excess stain was removed by rinsing with distilled water thereafter.

### Immunoblotting and immunoprecipitation

Cellular proteins were isolated by employing RIPA lysis buffer supplemented with a protease/phosphatase inhibitor cocktail. The concentration of the extracted proteins was determined using a BCA Protein Assay Kit. After boiling the protein lysates, they were separated via SDS-PAGE electrophoresis and subsequently transferred onto 0.22/0.45 μm PVDF membranes (Millipore). The membranes were blocked and then incubated with primary antibodies at 4 °C for 12 hours. Following three washes with PBS, the membranes were incubated with corresponding HRP-conjugated secondary antibodies, and the luminescent signals were detected and recorded using a Tanon gel imaging system.

The primary and secondary antibodies utilized in this study are listed as follows: β-catenin (dilution 1:1000, Catalog: 610154, BD Biosciences), UBA52 (dilution 1:1000, Catalog: ab109227, Abcam), HOPX (dilution 1:1000, Catalog: HPA030180, MilliporeSigma), Tubulin (dilution 1:100000, Catalog: T6074, MilliporeSigma), Actin (dilution 1:100000, Catalog: 4967, Cell Signaling Technology), APC (dilution 1:200, Catalog: OP44, MilliporeSigma), Axin1 (dilution 1:1000, Catalog: 2087S, Cell Signaling Technology), GSK3β (dilution 1:2000, Catalog: 12456S, Cell Signaling Technology), CK1 (dilution 1:1000, Catalog: 2655S, Cell Signaling Technology), HA (dilution 1:2000, Catalog: 11867431001, MilliporeSigma), Flag (dilution 1:2000, Catalog: 14793S, Cell Signaling Technology), Myc-tag (dilution 1:2000, Catalog: 2278S, Cell Signaling Technology), and His-tag (dilution 1:2000, Catalog: 12698, Cell Signaling Technology).

For the immunoprecipitation (IP) assays, cell lysates were incubated overnight with the designated primary antibody or a normal IgG control (dilution 1:100, Catalog: 3900S, Cell Signaling Technology). The resulting immune complexes were precipitated using protein A/G magnetic beads (Thermo Scientific). After thorough washing, the precipitated immune complexes were subjected to either western blot analysis or LC-MS analysis as required (Supplementary Table 3).

### Cell lines and plasmids

HEK293T, HCT116, and CT26 cell lines were cultured in Dulbecco's Modified Eagle Medium (DMEM, Invitrogen) supplemented with 10% fetal bovine serum (FBS). Transfection mixtures were prepared in advance as follows: each mixture contained 250 μl Opti-MEM, 10 μl Lipofectamine 2000, and either 10 μg plasmid DNA or 25 μM siRNA, which was then allowed to stand for 20 minutes. Plasmids were constructed using standard recombinant DNA manipulation techniques. The specific sequences of the siRNAs and plasmids employed in this study are provided in Supplementary Table 2.

### CRC cell isolation for flow cytometry and transcriptomic analysis

Minced fragments of colon tumors were gently rinsed in HBSS containing 1 mM DTT, and subsequently immersed in HBSS supplemented with 10 mM EDTA. This mixture was subjected to mechanical oscillation at 37 °C for 20 minutes, and the treatment was repeated twice. Afterward, intestinal epithelial cells present in the cell digestion mixture were harvested via centrifugation. The resulting single-cell suspension was subjected to sorting using the BD FACSAria Fusion Sorter (BD Biosciences), with sorting performed based on fluorescent signal detection. The sorted cell pellet was resuspended in pre-cooled PBS, after which cellular proteins were extracted. Alternatively, the sorted cells were resuspended in FACS buffer to facilitate subsequent cell analysis and RNA-Seq experiments.

### Statistical analysis

All the data in the graphs are presented as mean ± SD, statistical method and significance are reported in the Results and Figure Legends. The significance of the differences between groups was evaluated using analysis of two-tailed Student's *t*-test or one-way ANOVA, and *P* < 0.05 was considered significant (*: *P* < 0.05, **: *P* < 0.01, ***: *P* < 0.001). ns, no significance.

## Results

### High-iron diet enhances CRC tumorigenicity

**Figure 1:**
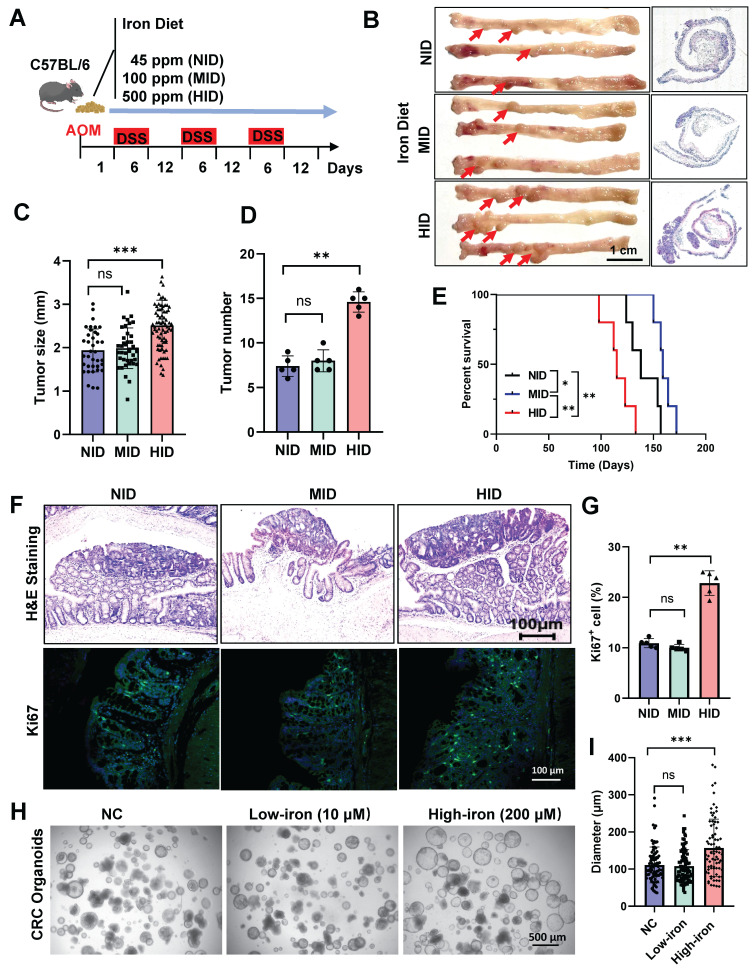
** High-iron diet enhances CRC tumorigenicity.** (A) Schematic of dietary exposure in AOM/DSS-treated mice. (B-D) Representative macroscopic images and histological tumor images (B), and tumor size quantification (C, D) of AOM/DSS-treated mice (n=5) fed three different diets. Scale bar, 1 cm. The red arrows point to tumors. (E) Survival of AOM/DSS-treated mice (n=5) fed three different diets. (F-G) Representative H&E and Ki67 staining (F), and quantification (G) of Ki67-stained colons in AOM/DSS-treated mice (n=5) fed three different diets. Scale bar, 100 µm. (H-I) Representative images (H) and quantification (I) of organoids from different iron-diet treated mice (n=5). Scale bar, 100 µm. All data are shown in mean ± SD and analyzed by one-way ANOVA. *: *P* <0.05. **: *P* <0.01. ***: *P* <0.001. ns, not significance.

To explicit the effect of dietary interventions on colon tumor formation, we designed diets which contain varying iron contents: NID: normal iron diet (45 ppm), MID: middle iron diet (100 ppm), HID: high iron diet (500 ppm) (Fig. 1A). We established a colitis-associated tumor mouse model by injecting azoxymethane (AOM) followed by three cycles of dextran sodium sulfate (DSS) in mice fed with different diets (Fig. 1A). Elevated Fe^3+^ levels were observed in high-iron treated tumor tissues using Prussian Blue Staining (Fig. S1A). High-iron diet robustly increased colon tumor formation, tumor size and tumor number in this orthotopic CRC (Fig. 1B-D), and high-iron fed mice showed significantly reduced long-term survival compared with MID and NID counterparts (Fig. 1E). To assess tumor proliferation, we quantified the number of Ki67^+^ cells within the intestinal tumor tissues: MID and NID did not significantly affect cell proliferation and high-iron diet robustly increased cancer cell proliferation (Fig. 1F, G). To examine the role of each dietary condition on ISCs, organoid system was performed to recapitulate the growth of the intestinal tumor derived from stem cells. Crypts derived from each dietary condition were equally plated and established. High-iron feeding enhanced the organoid-forming capacity of intestinal ISCs, such growth-promoting effects were not observed in MID and NID treated mice (Fig. 1H, I), indicating that the high-iron diet boosted the proliferation of crypt cells.

To determine whether iron was sufficient for enhancing tumor growth, CRC mice were treated with an iron chelator deferoxamine (DFO) (Fig. S1B). A decrease in the burden of adenocarcinoma was shown in mice treated with DFO (Fig. S1C-F). DFO treatment significantly decreased the proliferation of colon cells confirmed by Ki67 staining (Fig. S1G, H). Such growth-inhibition was also observed in organoids from DFO-treated CRC mice (Fig. S1I, J). Generally, these results indicate that a high-iron diet promotes colorectal tumor growth.

### High-iron enhances Hopx^+^ ISC function

**Figure 2:**
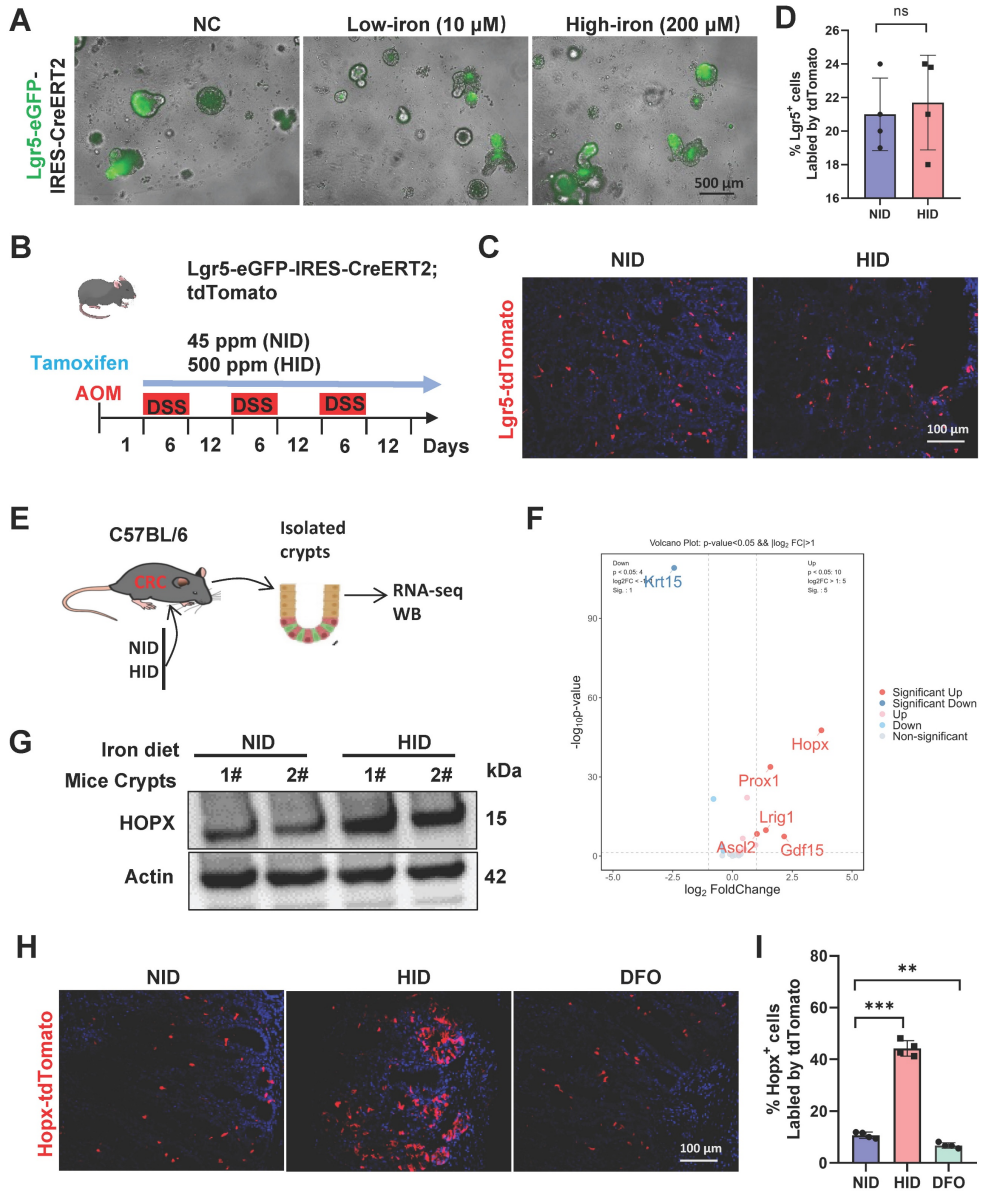
** High-iron enhances Hopx^+^ ISC function.** (A) Representative images of Lgr5-GRP cells of organoids from AOM/DSS-treated Lgr5-eGFP-IRES-CreERT2 mice (n=5) fed three different diets. Scale bar, 100 µm. (B) Schematic of dietary exposure in AOM/DSS-treated *Lgr5-eGFP-IRES-CreERT2;tdTomato* mice. (C-D) Representative images (C) and quantification (D) of Lgr5-positive colons in AOM/DSS-treated mice (n=4) fed different diets. Scale bar, 100 µm. (E) Experimental design to isolate crypt cells from CRC mice, then performed RNA-seq analysis. (F) Volcano plot analysis of intestinal stem cells markers in CRC mice (n=3) fed different diets. (G) Immunoblots for Hopx in crypts from AOM/DSS-treated mice fed different diets. (H-I) Representative images (H) and quantification (I) of Hopx-tdTomato cells of AOM/DSS-treated *Hopx^CreER^*;*Rosa^tdTomato^* mice (n=4) fed different diets. Scale bar, 100 µm. All data are shown in mean ± SD and analyzed by two-tailed Student's *t*-test (D) or one-way ANOVA (I). **: *P* <0.01. ***: *P* <0.001. ns, not significance.

Lgr5^+^ cancer cells capable of self-renewal and self-differentiation are served as stem cells in human CRC via lineage tracing, while selective ablation of Lgr5^+^ CSCs leads to tumor regression 27. We sought to determine the effect of iron on the tumorigenic potential of Lgr5 cells *in vitro*, Lgr5-eGFP-IRES-CreERT2 mice were induced as CRC models to culture organoids *ex vivo*. We observed that high-iron did not enhance the number of Lgr5-GFP^+^ cells in organoids compared with control (Fig. 2A, S2A). The lineage-tracing model of Lgr5 ISC (Lgr5-eGFP-IRES-CreERT2;Rosa26 Loxp-Stop-Loxp (LSL)-tdTomato) was performed to define the growing activity of ISC in response to iron *in vivo*, and tdTomato labelling in ISCs and their offspring was activated by tamoxifen administration. We then quantified the tdTomato-positive clones in CRC tissues after tamoxifen administration under normal and high-iron regimens (Fig. 2B). A minimal proportion of the tdTomato-labeled population emerged in colon tumor, and the frequency of the lineage-traced tdTomato^+^ progeny did not significantly increase in the tumor compared with controls after 54-day high-iron feeding, indicating that high-iron-treated Lgr5^+^ ISCs have not enhanced output (Fig. 2C, D). Overall, our data confirmed that the dispensable function of Lgr5^+^ ISCs in the CRC occur during tumor formation stimulated by high-iron diet to generate greater numbers of progeny in intestinal tumorigenesis.

To explore the mechanisms underlying iron-mediated tumor progression, RNA-seq was performed using colonic crypts from CRC mice fed with or without iron diet (Fig. 2E). Iron-treated cells exhibit significant changes in the expression of global genes (Fig. S2B). Among the intestinal stem cell genes which were mostly enriched following high-iron was Hopx (Fig. 2F), which has been identified to be beneficial for the damage-repair of intestinal mucosal 28. Therefore, we speculated that Hopx enrichment contributes to CRC growth promoted by high-iron feeding. We further identified whether Hopx enrichment is a result of high-iron feeding, increased expression of Hopx both in mRNA and protein levels was observed in high-iron treated CRC mice (Fig. 2G, S2C-E).

Hopx has been considered as a marker for slow-cycling stem cells located adjacent to Lgr5^+^ cells. These cells may function as a quiescent reservoir for Lgr5^+^ cells and have been linked to regeneration after colonic injury 15, 29. To visualize Hopx^+^ stem-cell dynamics after high-iron treatment during tumor formation, we inserted Cre-ERT2 into the Hopx locus and crossed it with Rosa26lsl-tdTomato to generate Hopx-CreERT2/Rosa-LSL-tdTomato (HopxCreERT2/RosaTd) mice to label Hopx^+^ cells for lineage-tracing experiments (Fig. S2F). To determine whether Hopx marks regenerative stem cells that contribute to re-establishing the intestinal epithelium after iron treatment in advanced CRC, HopxCreERT2/RosaTd CRC mice were injected with tamoxifen (Tam) to label Hopx^+^ cells and their offspring. Notably, the percentage of Hopx-tdTomato^+^ cells increased 5-fold after high-iron treatment, as determined by coimmunofluorescence (co-IF) analysis, whereas the tdTomato-labeled cells were observed sporadically in the non-iron-treated CRC tissues. Next, we found significant iron accumulation in Hopx^+^ cells with a high-iron diet (Fig. S2G), based on an Fe^3+^-specific fluorescent probe, this suggest that the expansion of Hopx^+^ cells due to high-iron is most likely driven by endogenous signals. Furthermore, such increase was also reversed by DFO in CRC mice (Fig. 2H, I). Thus, these findings demonstrate that high iron diet augments the regenerative capacity of Hopx^+^ intestinal stem cells (ISCs) to functionally contribute to colon tumor formation rather than Lgr5^+^ ISCs.

### Depletion of Hopx^+^ cells dramatically impairs CRC formation following iron-induced growth

**Figure 3:**
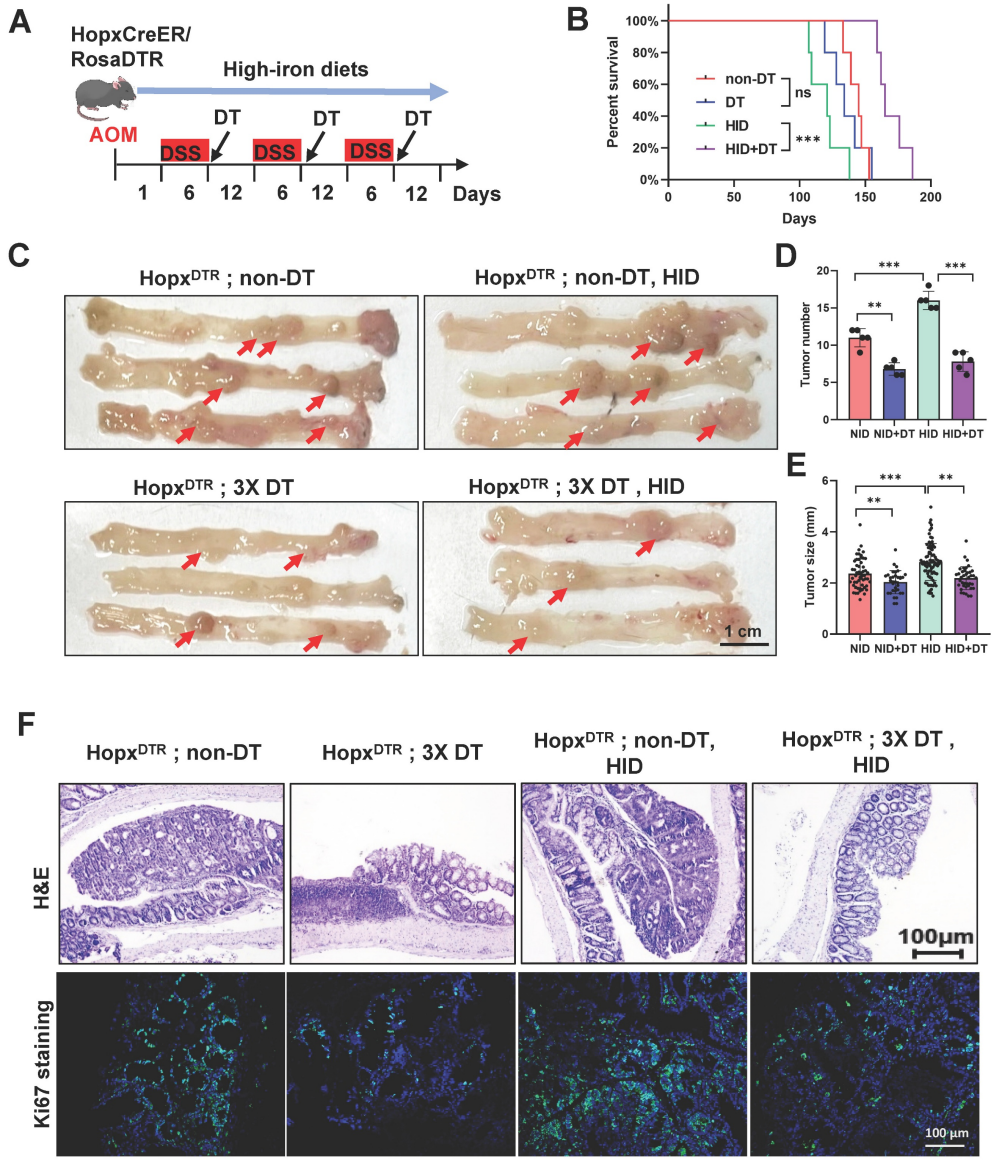
** Depletion of Hopx^+^ cells dramatically impairs CRC formation following iron-induced growth.** (A) Experimental scheme for Hopx^+^ cells ablation and iron diets. (B) Survival of AOM/DSS-inducted Hopx**^CreERT2^**;DTR mice (n=5) treated with different diets. (C-E) Representative macroscopic images (C), tumor size quantification (D) and tumor number quantification (E) of AOM/DSS-treated mice (n=5) treated as shown. Scale bar: 100 μm. Red arrows point to tumors. (F) Representative H&E and Ki67 staining from Hopx^CreERT2^;DTR mouse RC tissues (n=5) treated as shown. Scale bar: 100 μm. All data are shown in mean ± SD and analyzed by one-way ANOVA. **: *P* <0.01. ***: *P* <0.001. ns, no significance.

To assess whether the emerging Hopx^+^ stem cells exert a functional effect on tumor progression under high-iron dietary, we employed HopxCreER/RosaDTR mice to construct an AOM/DSS-induced CRC model (Fig. S3A). Subsequently, we ablated this specific cell population in the HopxCreER/RosaDTR mouse via diphtheria toxin (DT) injection, which was initiated at the time of each DSS withdrawal period (Fig. 3A). The depletion efficiency of Hopx^+^ cell was confirmed in the CRC tissues 7d after DT treatment (Fig. S3B). We found a significantly increased long-term survival in the combination mice compared with high-iron counterparts (Fig. 3B). The ablation of the Hopx-expressing population has a growth-inhibiting effect on tumor formation: Hopx;DTR mice treated with three doses of diphtheria toxin (DT) reveal a decrease in rectum tumor number and tumor size compared to untreated animals (Fig. 3C-E, S3C). Analysis of the tumors of Hopx;DTR animals 54 days following high-iron feeding (in the absence of DT) reveals an increase in tumor number and tumor size, however, this increase was significantly suppressed in mice treated with the combination of high-iron and DT (Fig. 3C-E, S3C). To assess proliferation, we quantified the number of Ki67^+^ cells within the intestinal tumor tissues and confirmed that Hopx^+^ cell ablation results in a marked attenuation of the regenerative response after DT in the high-iron feeding animals (Fig. 3F). Together, these findings establish an essential function of the emerging Hopx^+^ stem cells in promoting CRC generation in the setting of high-iron diet.

### Induction of Wnt pathway by iron requires Hopx

**Figure 4:**
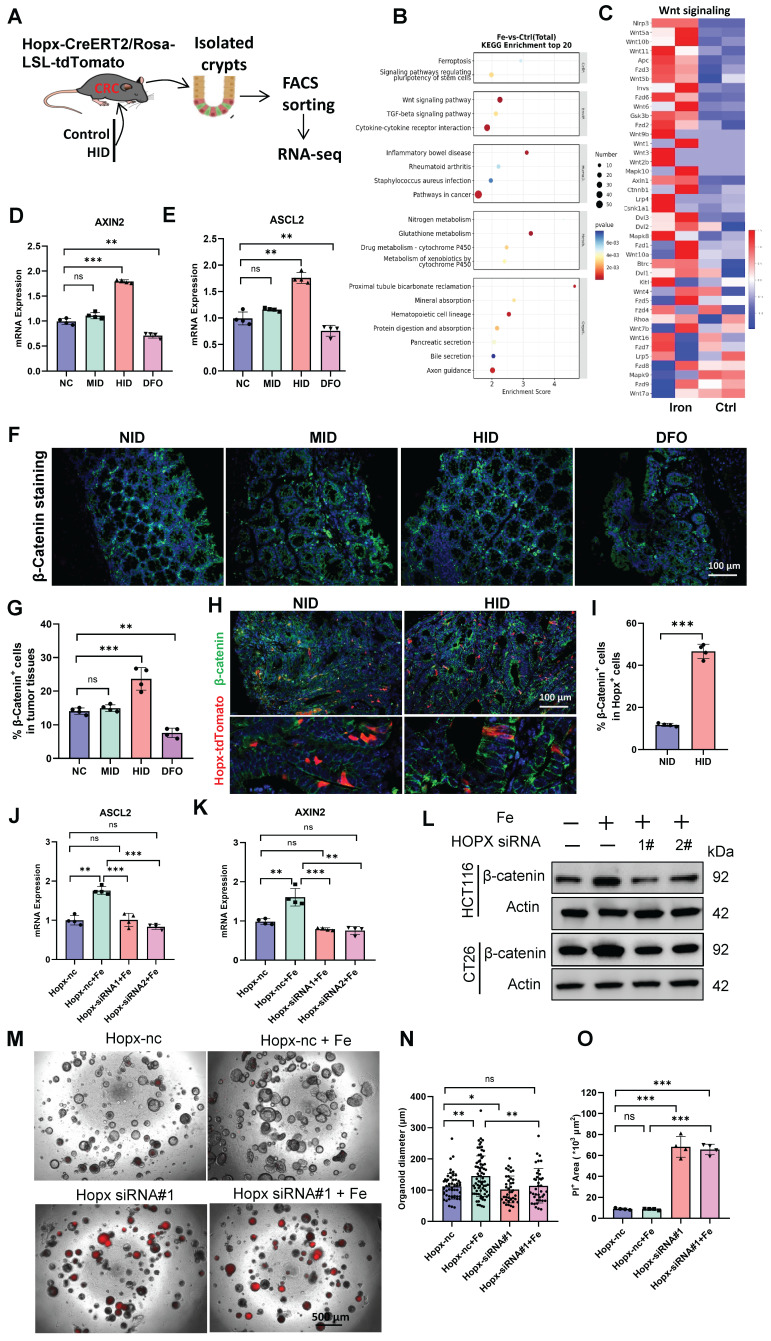
** Induction of Wnt pathway by iron requires Hopx.** (A) Experimental design to isolate Hopx-tdTomato cells using FACS in *Hopx^CreER^*;*Rosa^tdTomato^* CRC mice fed different diets, then performed RNA-seq analysis. (B-C) GO analysis and Heatmap of gene expression in FACS-sorted Hopx^+^ cells form CRC mice fed different diets. n = 2 replicates/time point. (D-E) qRT-PCR analysis of Wnt-pathway downstream targets of crypts from mice (n = 4) treated different iron diets. (F-G) Representative images (F) and quantification (G) of β-catenin staining in AOM/DSS-treated mice (n = 4) fed different diets. Scale bar: 100 μm. (H-I) Representative images (H) and quantification (I) of β-catenin staining in *Hopx^CreER^*;*Rosa^tdTomato^* CRC mice (n = 4) fed different diets. Scale bar: 100 μm. (J-K) qRT-PCR analysis of Wnt-pathway downstream targets in organoids treated as shown, n = 4. (L) Immunoblots for β-catenin in CRC cells treated as shown. (M-O) Representative images (M) and quantification (N, O) of PI Staining in organoids (n = 4) exposed to iron or/and Hopx siRNA. Scale bar: 500 μm. All data are shown in mean ± SD and analyzed by two-tailed Student's *t*-test (I) or one-way ANOVA (D, E, G, J, K, N, O). *: *P* < 0.05. **: *P* < 0.01. ***: *P* < 0.001. ns, no significance.

To gain mechanistic insight into how Hopx-activation mediated by high-iron augments cellular proliferation and colorectal tumorigenesis, we performed RNA sequencing analysis on FACS-sorted Hopx^+^ cells from HopxCreERT2/RosaTd CRC mice fed with or without high-iron diet (Fig. 4A). Several Wnt pathway and DNA replication responses were enriched in the cells, based on gene expression analysis (Fig. 4B, C, S4A-D). High-iron robustly induced expression of critical genes in the Wnt pathway at the mRNA and protein level in ISCs, and such expression was suppressed by iron chelator deferoxamine (Fig. 4D-G). Wnt signaling were detected in iron-exposed HopxCreERT2/RosaTd CRC tissues, and immunofluorescence staining clearly revealed that β-catenin activation was selectively upregulated in Hopx^+^ CSCs compared with that observed in normal iron-treated mice (Fig. 4H, I).

To determine whether the activation of Wnt signaling induced by high-iron required Hopx expression, we induced CRC in wild-type mice and then cultured tumor organoids. Remarkably, the functional knockdown of Hopx using siRNA strongly abated the activation of the Wnt pathway, which is activated by iron stimulation (Fig. 4J, K). Similar results were also observed at protein level in CRC cell lines (HCT116 and CT26) (Fig. 4L). The data revealed that high-iron strengthened the activation of Wnt signaling in a Hopx-dependent manner. We next performed a colony-formation assay and observed that high-iron did not enhance the organoid-forming capacity of ISCs following siRNA-mediated Hopx downregulation (Fig. 4M-O), confirming that Hopx was key to Wnt signaling mediated by iron in CRC growth.

### Hopx regulates the Wnt pathway by controlling the β-catenin level

**Figure 5:**
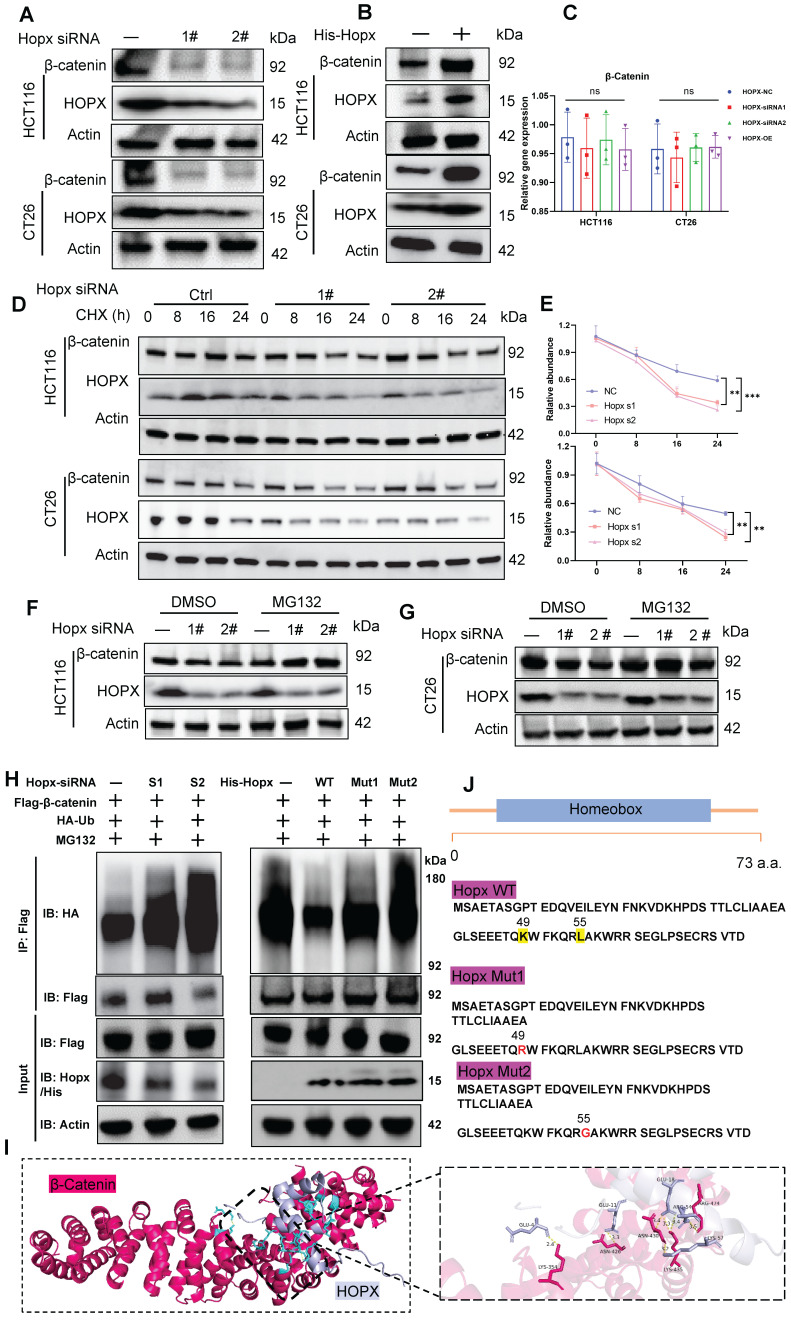
** Hopx regulates the Wnt pathway by controlling the β-catenin level.** (A-B) Immunoblots for Hopx in HCT116 and CT26 cells with Hopx knockdown or transfected with His-Hopx. n=3. (C) mRNA levels of β-catenin in HCT116 and CT26 cells with Hopx knockdown or transfected with His-Hopx. n=3. (D-E) Representative images (D) and quantification (E) of protein level of β-catenin in HCT116 and CT26 cells with or without Hopx knockdown following CHX treatment (100 µg/mL) for the indicated times. n=3. (F-G) Representative images of protein level of β-catenin in HCT116 and CT26 cells with or without Hopx knockdown following MG132 treatment (10 µM, 6 h) for the indicated times. n=3. (H) Denaturing IP with an anti-Flag antibody and IB of HA-Ub, Flag-β-catenin, Hopx/His-Hopx and Actin in HEK293T cells transfected with the indicated siRNA or plasmids following MG132 treatment (10 µM, 6 h). n=3. (I) Structure docking to simulate the interaction between Hopx and β-catenin. (J) Hopx domain structure and mutants used in the study (WT: His-Hopx-WT; Mut1: His-Hopx-L49R; Mut2: His-Hopx-L55G). All data are shown in mean ± SD and analyzed by one-way ANOVA (C, E). **: *P* <0.01. ***: *P* <0.001. ns, no significance.

To further understand whether Wnt activation is dependent on Hopx expression, we first examined whether Hopx affects the Wnt signaling pathway. We found that the expression levels of Wnt target genes (ASCL2 and AXIN2) were significantly activated by Hopx upregulation in CRC organoids, and such upregulation was reversed by the knockdown of Hopx using siRNA (Fig. S5A, B). These data demonstrate that Hopx positively regulates the Wnt signaling pathway. β-catenin has been identified to be a marker of the activation of Wnt-signal pathway 30. We then examined the relationship between Hopx and β-catenin in the Wnt pathway. Knockdown of Hopx dramatically reduced β-catenin protein expression, whereas overexpression of Hopx elevated β-catenin protein expression in CRC cells (Fig. 5A, B). However, the mRNA expression level of β-catenin did not significantly change with either knockdown or overexpression of Hopx (Fig. 5C). Correspondingly, after treatment with cycloheximide (CHX), Hopx knockdown facilitated but Hopx overexpression suppressed the degradation of endogenous β-catenin (Fig. 5D, E), suggesting that Hopx prolongs the half-life of the β-catenin protein. These findings demonstrate that Hopx inhibits β-catenin degradation in CRC cells.

The ubiquitin-proteasome and autophagy-lysosome systems are the two primary mechanisms for protein degradation 31. To study which degradation pathway drives Hopx-mediated β-catenin degradation, CRC cells were exposed to a proteasome inhibitor (MG132) or a lysosome inhibitor (chloroquine; CQ). MG132, but not CQ, reversed the reduction in β-catenin protein levels caused by Hopx knockdown (Fig. 5F, G, S5C, D), confirming that Hopx-mediated degradation of β-catenin occurs through the ubiquitin‒proteasome pathway. We then examined the effect of Hopx on the ubiquitination of β-catenin and found that the level of β-catenin ubiquitination was largely augmented by Hopx knockdown in HEK293T cells. However, Hopx overexpression inhibited the polyubiquitination of β-catenin (Fig. 5H). Molecular docking analysis shows that Hopx may bind with β-catenin protein (Fig. 5I). Hopx protein amino acid sequence with the mutations in key residues (49: lysine, 55: leucine) responsible for protein binding 32. To further figure out the effect of Hopx on the ubiquitination of β-catenin, First, we constructed His-Hopx-WT, His-Hopx-L49R (mutation at lysine 49 by arginine) and His-Hopx-L55G (mutation at leucine 55 by glycine) plasmids (Fig. 5J), we then co-expressed Flag-β-catenin, HA-ubiquitin (HA-Ub), His-Hopx-WT, His-Hopx-L49R or His-Hopx-L55G in HEK293T cells. Expression of His-Hopx WT, but not its mutant, prominently inhibited the ubiquitination of β-catenin (Fig. 5H). These results indicate that Hopx upregulates β-catenin via affecting ubiquitin-mediated degradation.

### Hopx is involved in the ubiquitination of β-catenin via UBA52

**Figure 6:**
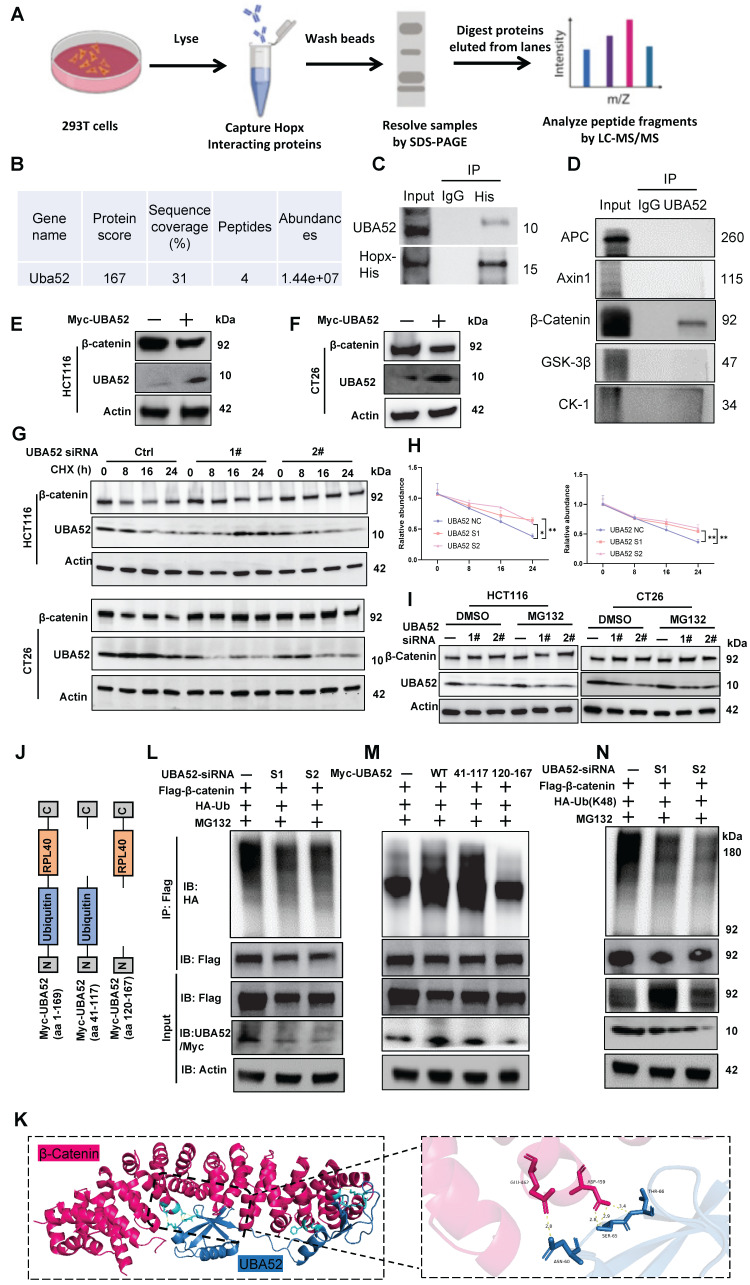
** Hopx is involved in the ubiquitination of β-catenin via UBA52.** (A) Schematic workflow for Hopx immunoprecipitation from HEK293T lysates followed by mass spectrometry (Hopx IP-MS) to identify Hopx interactors. (B) The mass spectrometry results identify UBA52 as a potential binding partner of Hopx. (C) Immunoprecipitation (IP) with an anti-His antibody and immunoblot analysis (IB) of UBA52 or His expression in HEK293T cells. (D) β-catenin and other major components of β-catenin destruction complex are co-immunoprecipitated with UBA52 in the co-immunoprecipitation assay. n=3. (E-F) Immunoblots for β-catenin in HCT116 and CT26 cells transfected with Myc-UBA52. n=3. (G-H) Representative images (G) and quantification (H) of protein level of β-catenin in HCT116 and CT26 cells with or without UBA52 knockdown following CHX treatment (100 µg/mL) for the indicated times. n=3. (I) Representative images of protein level of β-catenin in HCT116 and CT26 cells with or without UBA52 knockdown following MG132 treatment (10 µM, 6 h) for the indicated times. n=3. (J) UBA52 domain structure and deletion mutants used in the study as shown. (K) Structure docking to simulate the interaction between UBA52 and β-catenin. (L-M) Denaturing IP with an anti-Flag antibody and IB of HA-Ub, Flag-β-catenin, UBA52/Myc-UBA52 and Actin in HEK293T cells transfected with the indicated siRNA or plasmids following MG132 treatment (10 µM, 6 h). (N) Denaturing IP (with an anti-Flag antibody) and IB of HA, Flag, UBA52 and Actin in HEK293T cells transfected with the indicated plasmids following MG132 treatment (10 µM, 6 h). n=3. All data are shown in mean ± SD and analyzed by one-way ANOVA (H). *: *P* <0.05. **: *P* <0.01.

Although Hopx suppresses ubiquitination degradation of β-catenin, Hopx does not belong to any of the ubiquitination or deubiquitinating enzyme families. Therefore, we speculate that there may be other ubiquitin proteins involved in the inhibition of β-catenin degradation by Hopx protein. We next sought to determine whether Hopx can interact with a binding partner to stabilize β-catenin. Encouragingly, we found that UBA52, a ubiquitin-specific protease belonging to the ubiquitinating enzyme family, was among the most common potential interacting proteins of Hopx according to IP-MS results (Fig. 6A, B, S6A, B). Further co-IP assays confirmed that Hopx could interact with endogenous UBA52 (Fig. 6C). A cytoplasmic destruction complex including (Axin: central scaffold protein, APC: adenomatous polyposis coli, GSK3: glycogen synthase kinase-3, CK1: casein kinase 1) is critical for β-catenin regulation 33. Cytosolic β-catenin captured by the destruction-complex was then degraded by the ubiquitin ligase in the absence of Wnt signaling. To address whether UBA52 is involved in Hopx-mediated β-catenin degradation-suppression, immunoprecipitation was used to examine the relationship between UBA52 and the β-catenin destruction complex in HEK293T cells. Co-IP assays confirmed that UBA52 could directly interact with β-catenin rather than any key components of the β-catenin destruction complex (Fig. 6D). We conducted reverse co-immunoprecipitation targeting β-catenin. Co-IP analysis revealed an interaction between Hopx and β-catenin as well as between UBA52 and β-catenin (Fig. S6C, D).

Next, we examined whether UBA52 affects the stability of β-catenin. Unexpectedly, overexpression of UBA52 led to decreased protein levels of β-catenin in CRC cells (Fig. 6E, F). Additionally, the half-life of the β-catenin protein was significantly longer in UBA52-knockdown cells (Fig. 6G, H). Further investigation confirmed that MG132 treatment restored the level of β-catenin following UBA52 knockdown (Fig. 6I).

Structurally, UBA52 consists of a ubiquitin domain and a ribosomal protein L40 (Fig. 6J), which is essential for both the protein-degradation by the ubiquitin-proteasome pathway and ribosome assembly 34. Molecular docking analysis shows that UBA52 may bind with β-catenin protein (Fig. 6K). We then figured out the role of UBA52 in the ubiquitination of β-catenin and found that knockdown of UBA52 decreased the K48-linked, instead of K63-linked, polyubiquitination of β-catenin (Fig. 6L, N, S6E). Consistent with these findings, overexpression of UBA52 increased the ubiquitination of β-catenin (Fig. 6M). Previous study showed that UBA52 may function as a potential E3 ubiquitin ligase 35. As shown in Fig. S6F, *in vitro* ubiquitination assay is consistent with the hypothesis that UBA52 can induce the ubiquitination and degradation of β-catenin without any E3 ligase. We then addressed which domains of UBA52 interact with β-catenin protein using different regions of truncated UBA52 fragments, the aa 41-117 of UBA52 were confirmed as a key domain for β-catenin ubiquitylation (Fig. 6M), indicating that the ubiquitylase activity of UBA52 is essential for β-catenin ubiquitylation. In summary, these findings demonstrate that UBA52 decreases the stability of β-catenin by promoting its K48-linked polyubiquitination.

### HOPX competitively binds to UBA52 to control UBA52-mediated polyubiquitination degradation of β-catenin

**Figure 7:**
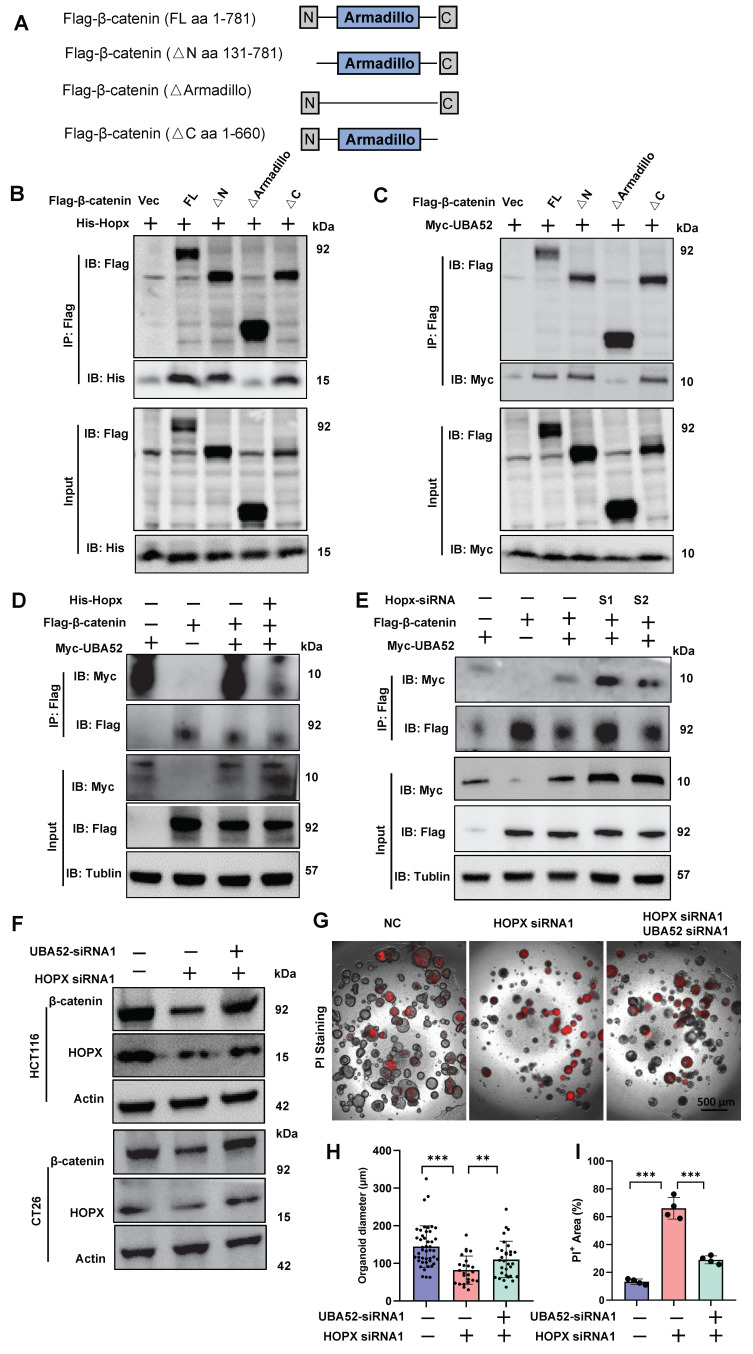
** HOPX competitively binds to UBA52 to control UBA52-mediated polyubiquitination degradation of β-catenin.** (A) β-catenin domain structure and deletion mutants used in the study. (B) Molecular characterization of Hopx interaction with different truncated fragments of β-catenin in HEK293T cells. Transfected HEK293T cell lysates were subjected to immunoprecipitation (IP). Input lysates and IP samples were subjected to immunoblotting (IB). (C) Molecular characterization of UBA52 interaction with different truncated fragments of β-catenin in HEK293T cells. Transfected HEK293T cell lysates were subjected to immunoprecipitation (IP). Input lysates and IP samples were subjected to immunoblotting (IB). (D-E) The interaction between β-catenin and UBA52 following the overexpression of Hopx (D) or the knockdown of Hopx (E) was examined in HEK293T cells by co-immunoprecipitation. (F) Immunoblots for β-catenin in HCT116 and CT26 cells following the knockdown of Hopx or/and UBA52. (G-I) Representative images (G) and quantification (H, I) of PI Staining in organoids (n=4) following the knockdown of Hopx or/and UBA52. Scale bar: 500 μm. All data are shown in mean ± SD and analyzed by one-way ANOVA (H, I). **: *P* <0.01. ***: *P* <0.001.

Based on the structure-function findings, we hypothesized that Hopx would suppress β-catenin ubiquitination mediated by UBA52 via competing with UBA52 for interacting with β-catenin. The central repeat region, consisting of 12 Armadillo repeats, is the main region that interacts with other proteins. Deletion mapping experiments of β-catenin were performed in a heterologous system to identify the β-catenin region that binds Hopx and UBA52 (Fig. 7A). HEK293T cells were transfected with epitope-tagged Hopx or UBA52 along with β-catenin deletion mutants. We observed that both Hopx and UBA52 bind to the Armadillo region of β-catenin by co-immunoprecipitation experiments using diverse transfected cells (Fig. 7B, C), consistent with a possible competitive binding model.

We further tested whether Hopx expression affects the interaction between UBA52 and β-catenin. The binding of UBA52 to β-catenin was inhibited by Hopx overexpression in HEK293T cells (Fig. 7D). By contrast, the knockdown of Hopx enhanced their interaction assessed by co-immunoprecipitation (Fig. 7E). In the context of CRCs, we found that the suppression of β-catenin protein mediated by Hopx knockdown was inverted by simultaneous knockdown of UBA52 (Fig. 7F). The results of *in vitro* protein competition experiments revealed that increasing amounts of Hopx levels decreased β-catenin-UBA52 complex formation (Fig. S6G), overall consistent with a competitive binding model. Consistent with these functional experiments, Hopx knockdown inhibited the capacity of self-renewal in CRC organoids, and such inhibition was inverted by UBA52 knocked down with siRNA (Fig. 7G-I). In summary, these findings suggest that Hopx expression attenuates the interaction between UBA52 and β-catenin, thus directly disrupts UBA52-mediated β-catenin ubiquitination, resulting in maintaining the protein levels of β-catenin and CRC biologic phenotypes.

### Iron levels correlate with Hopx-Wnt activity in Human CRCs

**Figure 8:**
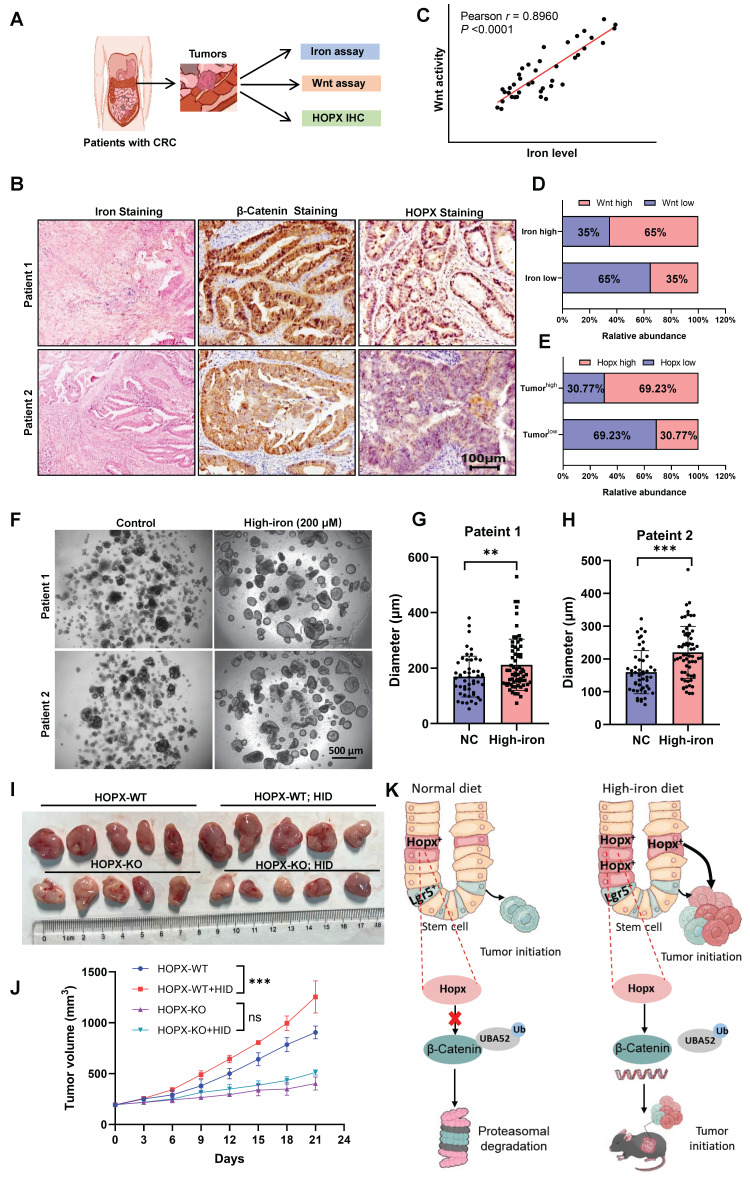
** Iron levels correlate with Hopx-Wnt activity in Human CRCs.** (A) Schematic showing steps involved in studying the correlation between Wnt activity, HOPX and iron levels in colorectal cancer. n=40. (B) Representative images of iron (PB) staining (left), β-catenin staining (middle) and HOPX staining of CRC tumor sections from two patients. Scale bar: 100 μm. (C) Correlations between Wnt activity and iron levels in colorectal cancer (n=40). (D) Quantification of Wnt activity of tumor (n=40). Tumors were segregated into iron-low (indicated as Iron^Low^) and iron-high (indicated as Iron^High^) groups. (E) Quantification of HOPX of tumor in iron levels for tumors (n=40). Tumors were segregated into Tumor^High^ (samples with high Wnt and high iron levels) and Tumor^Low^ (samples with low Wnt and low iron levels) groups. (F-H) Representative images (F) and quantification (G, H) of human CRC organoids (n=4) exposed to different iron concentrations. Scale bar: 500 μm. (I-J) Representative images (I) and quantification (J) of subcutaneous AK-HOPX^WT^ or AK-HOPX^KO^ human CRC tumors in mice (n=5) following 15 days iron-diet. (K) Schematic showing how high-iron diet alters Hopx^+^ ISC activity to promote CRC tumorigenic. All data are shown in mean ± SD and analyzed by two-tailed Student's *t*-test (G, H) or one-way ANOVA (J). **: *P* <0.01. ***: *P* <0.001. ns, no significance.

We next investigated the correlation between iron-level and Wnt activity in human CRC tissues. Wnt activity was measured using β-catenin IHC staining and iron levels were simultaneously measured by Prussian blue (PB) staining in tumor tissues (Fig. 8A). This cohort (40 samples) was divided into high-iron (20 samples) and low-iron groups (20 samples) based on the median of iron levels (the percentage of Prussian blue-positive cells). Strikingly, 13 samples belonging to high-iron group also showed high Wnt activity (Fig.8B, D, S7A). These findings suggested that iron level is significantly positive with Wnt activity (Fig. 8C). Based on these results that Hopx plays a critical role in iron-mediated Wnt/β-catenin induction, we analyzed Hopx levels in tumor tissues, which iron levels positively correlate with Wnt signaling activity (Fig. 8B). We found increased levels of iron and Hopx in CRC tissues based on immunohistochemistry (Fig. 8B). Increased Hopx expression was shown in 80% CRC tissues which measured with higher positive for iron, compared with low-iron tissues (Fig. 8B). Tumor samples were hence divided into Tumor^High^ (high iron-levels and high Wnt) and Tumor^Low^ (low-iron levels and low Wnt) groups. Meanwhile, 69.2% samples of Tumor^High^ group have relatively increased expression of Hopx (Fig. 8E). Furthermore, GEPIA 2.0 database analysis showed a significant correlation between Hopx and β-catenin proteins in CRC (Fig. S7B). These findings highlighted that the detected correlation of iron and Hopx with Wnt pathway in CRC has functional relevance.

Furthermore, we examined whether the iron-Hopx axis is similarly effective in strengthening epithelial proliferation of human colon. Indeed, iron enhanced the growth of organoids in patients with CRC (Fig. 8F-H). To further evaluate the function of Hopx in iron-mediated growth-promotion of human colorectal cancer *in vivo*, human CRC organoids were transduced with APC^min/+^ and K-RAS^G12D^ forms (AK organoid), then AK organoid cells concomitantly expressing CRISPR/Cas9-mediated HOPX knockout were subcutaneously injected to generate xenograft tumors (Fig. S7C). HOPX knockout robustly blocked the growth of xenograft tumors which enhanced by iron diet (Fig. 8I, J). We further analyzed the prognostic value of HOPX in human CRC patients. Kaplan-Meier (KM) curves revealed that lower HOPX expression was linked to better overall survival (OS) rates in CRC patients (Fig. S7D). These data demonstrate that iron-mediated Wnt activation emerges in the context of high Hopx levels, further study of the association between iron and Hopx-Wnt activation would comprehensively elucidate the mechanism of the effect of iron in intestinal tumors.

## Discussion

This work has clarified that high-iron diet drives a unique cellular program of colon tumorigenesis distinct from normal diet 1, 36. A subset of self-renewing cancer cells can fuel tumor growth based on the cancer stem cell (CSC) notion. Previous studies have shown that the intestinal environment after high-iron diet treatment may be detrimental to epithelial cell repair or barrier recovery after injury 36. However, when the stem cell pool was disturbed by an injury, iron is critical for the cells responsible for re-establishing this compartment to drive the proliferative regeneration program. Here we propose that high-iron feeding enhances the intestinal epithelial renewal activity of ISCs. First, the regenerative capacity of high-iron-fed ISCs exceeds that of the normal treatment after injury. Second, high-iron stimulates Hopx-CTNNB1 signaling in ISCs. Third, Hopx competitively binds to UBA52 to control UBA52-mediated polyubiquitination degradation of β-catenin (Fig. 8K). Together, these results manifest that high-iron primes ISCs to robustly regenerate intestinal epithelium upon nutritional stimulation.

Recognizing which stem cell subpopulation executes the regeneration programs is key to insight into the role of iron on epithelial repair in the setting of chronic injury. In the mouse intestine, Lgr5^+^ stem cells are required for regeneration in a radiation injury model. In contrast, during colonic inflammation, they are dispensable for repair 37, raising a question as to the identity of colonic stem cells mediating iron-associated regeneration. Previous study proposed that Lgr5^+^ ISCs could be growth-inhibited by high-iron diet and control their transformation into secretory cells phenotype. In addition, iron overload can inhibit the Notch signaling pathway by increasing the oxidative stress and ferroptosis in intestinal stem cells 38. Our data show that Lgr5^+^ cells are dispensable for malignant hyperproliferation following iron-mediated robust intestinal regeneration. Here, our findings establish a prominent function of the emerging Hopx^+^ stem cells in iron-mediated growth-promotion of crypt regeneration in the setting of colitis. The link between cell cycle and metabolism regulation is associated by a notable increase in lipid synthesis for the synthesis and division of membrane organelles during G2 39, we therefore can easily speculate that Lgr5^+^ cells in a prolonged G2 phase may have higher lipid content and are more susceptible to ferroptosis induced by uncontrolled iron-overload. However, slow-cycling cells are characterized by low mitochondrial and metabolic activity, including ROS production 40, this relatively dormant condition likely enables ISCs to persist in a high-iron niche. Meanwhile, functional studies found Hopx served as an inhibitor of adipogenesis through suppressing de novo synthesis of lipids, protecting slow-cycling cells from lipid peroxidation and ferroptosis.

Hopx is thought to play a critical role in mammalian development and differentiation, it can inactivate the GATA6/Wnt7 pathway through directly interacting with HDAC2 41. Hopx exhibits low expression in tumor tissues, which is predominantly epigenetic suppression in some cancers, and the expression level of Hopx would be used as a potential prognostic marker for colorectal cancer patients. Defects of Hopx expression modulated by itself in an epigenetic manner leads to a loss of tumor suppressor and differentiated phenotypes 42. These findings suggest that Hopx can critically regulate epigenetic dynamics and be involved in the determination of human cell differentiation. However, our functional studies of HOPX overexpression or knockdown observed that induction of Wnt/β-catenin signaling by iron requires Hopx. These conflicts indicate the duo-functions of Hopx for the proliferation and differentiation of colon cancer in different contexts and emphasize the significance of Hopx in balancing the dynamics of cell growth and differentiation in various tissues. Future research will be needed to ascertain the function of Hopx in CRC tumorigenesis and progression.

The Wnt signaling pathway plays a critical role in maintaining and regulating ISCs 43. Previous studies have shown that iron could upregulate WNT signaling, and this upregulation can be effectively inhibited by iron chelation 30. Therefore, we figured out whether WNT activation is the key mechanism in iron-driven ISC expansion. We explored the potential mechanisms of ISC differentiation and self-renewal by detecting the expression of genes related to the Wnt signaling pathway. Our findings demonstrated that high-iron treatment boost ISC numbers and function by upregulating Wnt/β-catenin signaling in the Hopx^+^ stem cells. How do iron lead to the expansion of Hopx+ stem cell subpopulation, our previous study found that iron can induce the activation and nuclear translocation of Stat3 protein, and activated Stat3 protein can translocate into the nucleus and bind to the specific binding sites within the promoter region of the Hopx gene, thereby initiating and enhancing the transcriptional activity of the Hopx gene 44.

Under normal conditions, β-catenin phosphorylated by the destruction complex can be subsequently ubiquitinated and degraded to prevent the continuous activation of the Wnt signaling pathway 45. Moreover, several E3 ubiquitin ligases also degrade β-catenin protein such as SHPRH, SIAH1, and GID complexes 46, 47, suggesting that the β-catenin degradation mechanism is complex. In this study, we showed that Hopx affected the levels of β-catenin ubiquitination in colon cancer. Mechanistically, we found that Hopx non-catalytically blocks β-catenin ubiquitination by dampening the interaction between β-catenin and UBA52. UBA52 protein consists of a ubiquitin domain at the amino terminus and an RPL40 domain at the carboxyl terminus, which could be cleaved into RPL40 and ubiquitin 48, 49. This suggests that the adequate ubiquitin pool provided by UBA52 accelerates the degradation of β-catenin mediated by the proteasome. Previous studies have shown that UBA52 plays an important role in embryonic development and can drive colorectal cancer tumorigenesis 50, 51. In the present study, Hopx overexpression significantly rescued UBA52-mediated β-catenin degradation. In addition, the results from co-IP and MS showed that UBA52 interacts with both Hopx and β-catenin in the same region. Therefore, targeting the regulation of Hopx protein expression and function has emerged as an alternative strategy to interrupt the oncogenic functions of β-catenin.

In summary, this work indicated that high-iron could enhance ISCs proliferation through an iron-Hopx-Wnt signaling axis. Our study expands the concept of dietary patterns associated with CRC growth-promotion by demonstrating that iron contributes to the regeneration of the intestinal epithelium in a way that is unique to a normal diet. Notably, prospective randomized controlled studies in human cohorts are required to insight better how the proportion of iron intake augments ISC functions without increasing tumor risk before specific recommendations for prevention and treatment can be made. The iron-regulated signaling might act synergistically with surgery, chemotherapy or immunotherapy and might emerge as a new pillar of 'cancer metabotherapy'.

## Supplementary Material

Supplementary figures and tables.

## Data Availability

The authors confirm that the data supporting the findings of this study are available within the article and its supplementary materials.
