# Supplementary material for: Competitive Binding of UBA52 and HOPX Modulates β-catenin Stability in Colorectal Cancer in the Context of High-Iron Intake
Source: Int J Biol Sci. 2026 Apr 16;22(9):4564–83. doi: 10.7150/ijbs.126038 (PMC13182247; doi:10.7150/ijbs.126038)
Supplement: Supplementary file 1 — Supplementary figures and tables. [file ijbsv22p4564s1.pdf]

Supplementary Figure legends

Figure S1. High-iron diet enhances CRC tumorigenicity.

(A) Representative images of iron (Prussian blue reaction) staining of AOM/DSS-treated mice (n=5) fed three different diets. Scale bar, 100 μm. (B) Schematic of DFO (100 mg/kg) exposure in AOM/DSS-treated mice (n=5). (C-F) Representative macroscopic images (C) and histological tumour images (D), and tumour size quantification (E, F) of AOM/DSS-treated mice (n=5) fed three different diets. Scale bar, 1cm. (G-H) Representative H&E and Ki67 staining (G), and quantification (H) of Ki67-stained colons in and AOM/DSS-treated fed three different diets. Scale bar, 100 μm. (I-J) Representative images (I) and quantification (J) of organoids from iron-diet or DFO treated mice (n=5). Scale bar, 100 μm. All data are presented by mean ± SD and analyzed by two-tailed Student's *t*-test. *: P <0.05. **: P <0.01. ns, not significance.

Figure S2. High-iron enhances Hopx$^+$ ISC function

(A) Quantification of Lgr5-GRP cells of organoids from AOM/DSS-treated Lgr5-eGFP-IRES-CreERT2 mice (n=5) treated with different iron. (B) Heatmap of gene expression in AOM/DSS-treated mice (n=3) fed different diets. (C) qRT-PCR analysis of Hopx mRNA levels in tumor tissues (n=3) treated different iron diets. (D-E) Representative images (D) and quantification (E) of Hopx staining in AOM/DSS-treated mice (n=5) fed different diets. Scale bar, 100 μm. (F) Schematic showing the crossing between Hopx^{CreERT2} and Rosa^{tdTomato} mice. (G) Iron fluorescence staining of Hopx^{CreERT2}Rosa^{tdTomato} CRC mice (n=4)

treated with or without high-iron diet. Scale bar, 100 μm. All data are presented by mean ± SD and analyzed by one-way ANOVA. **: *P* <0.01. ***: *P* <0.001. ns, not significance.

Figure S3. Depletion of Hopx^{+} cells dramatically impairs CRC formation following iron-induced growth

(A) Schematic showing the crossing between Hopx^{CreERT2} Rosa^{tdTomato} mice and DTR mice. (B) Representative images of Hopx-tdTomato cells of AOM/DSS-treated Hopx^{CreER};Rosa^{tdTomato};DTR mice after DT treatment (7d). Scale bar, 100 μm. (C) Representative histological tumour images of AOM/DSS-treated Hopx^{CreER};Rosa^{tdTomato};DTR mice (n=5) fed different diets.

Figure S4. Induction of Wnt pathway by iron requires Hopx

(A-B) KEGG-based analysis and GO analysis of gene expression of FACS-sorted Hopx^{+} cells form CRC mice (n=2) fed different diets. (C-D) GSEA-based analysis of DNA replication and Wnt siginaling pathway in FACS-sorted Hopx^{+} cells form CRC mice (n=2) fed different diets.

Figure S5. Hopx regulates the Wnt pathway by controlling the β-Catenin level

(A-B) qRT-PCR analysis of Wnt-pathway downstream targets in organoids with Hopx knockdown or transfected with His-Hopx (n=3). (C-D) Representative images of protein level of β-Catenin in HCT116 and CT26 cells with or without Hopx knockdown following QC treatment (20 μm, 6 h) for the indicated times.

Figure S6. Hopx is involved in the ubiquitination of β-catenin via UBA52

(A) Flag-Hopx or vector-transfected HEK293T cells were subjected to immunoprecipitation with an anti-Flag antibody, followed by SDS–PAGE and Coomassie Blue Fast staining of proteins. (B) Top20 proteins as a potential binding partner of Hopx identified by the mass spectrometry. (C) Immunoprecipitation (IP) with an anti-Flag antibody and immunoblot analysis (IB) of Hopx or Flag expression in HEK293T cells. (D) Immunoprecipitation (IP) with an anti-Flag antibody and immunoblot analysis (IB) of UBA52 or Flag expression in HEK293T cells. (E) Denaturing IP (with an anti-Flag antibody) and IB of HA, Flag, UBA52 and Actin in HEK293T cells transfected with the indicated plasmids following MG132 treatment (10 μm, 6 h). (F) Recombinant β-Catenin proteins were subjected to in vitro ubiquitination assay in the absence or presence of in vitro-translated wild-type UBA52, and western immunoblotting with indicated antibodies. (G) Recombinant β-Catenin proteins and UBA52 proteins were subjected to in vitro competitive binding assays in the absence or presence of purified His-Hopx, and western immunoblotting with indicated antibodies.

Figure S7. Iron levels correlate with Hopx-Wnt activity in Human CRCs

(A) Representative images of iron (PB) staining (left), β-Catenin staining (middle) and HOPX staining of CRC tumor sections from two patients. Scale bar: 100 μm. (B) Correlations between HOPX expression and β-catenin

expression using human CRC tissues in the GEPIA 2.0 database. (C) CRISPR–Cas9 strategy used to generate mutant organoid lines (A: $APC^{min/+}$; K: $KRAS^{G12D}$). (D) Correlations between HOPX expression and overall survival (OS) using human CRC tissues in the GEPIA 2.0 database.

Figure S1

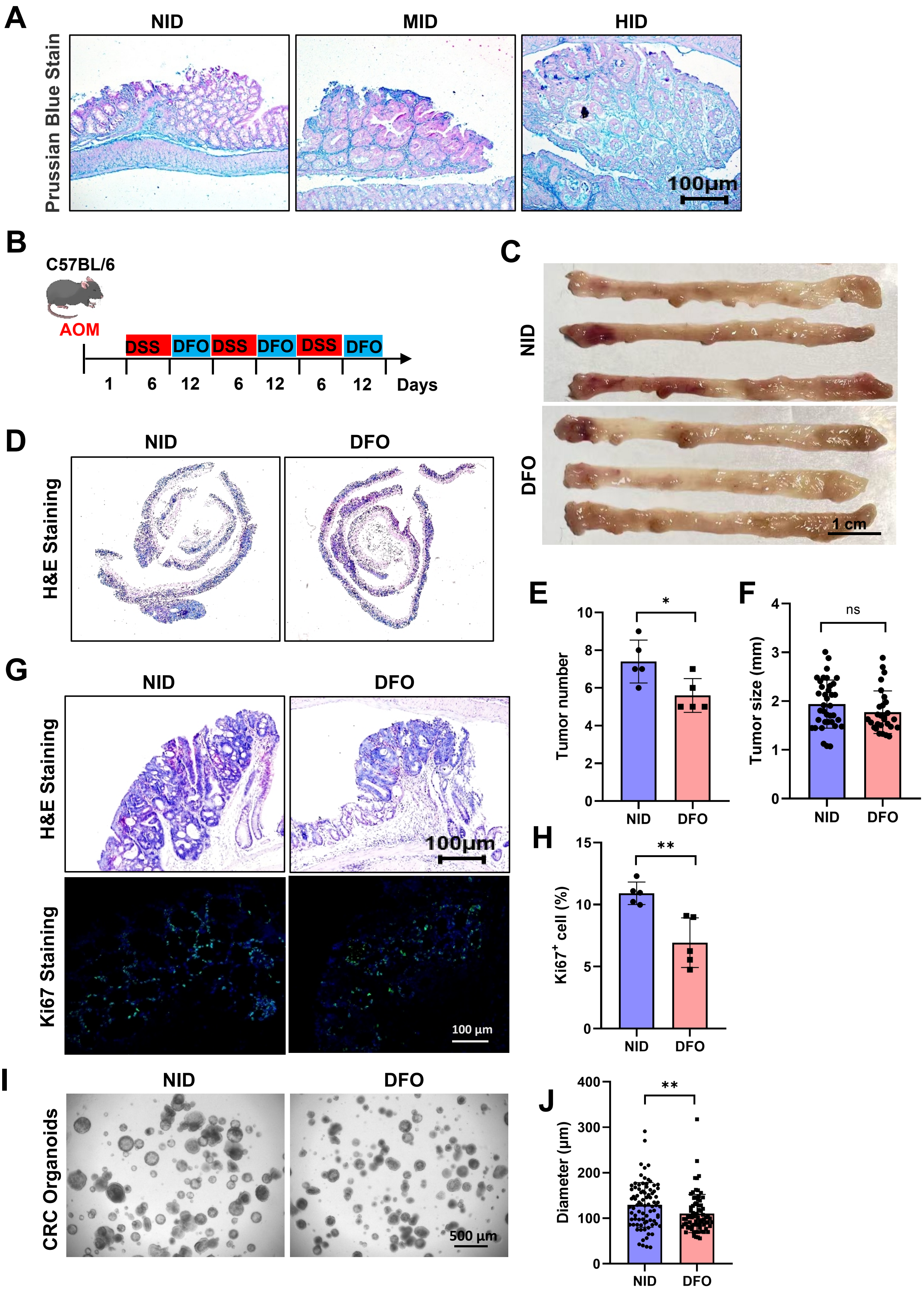

A
NID
MID
HID
Prussian Blue Stain
100μm
B
C57BL/6
AOM
DSS
DFO
DSS
DFO
DSS
DFO
1
6
12
6
12
6
12
Days
C
NID
DFO
1 cm
D
NID
DFO
H&E Staining
E
Tumor number
*
NID
DFO
F
Tumor size (mm)
ns
NID
DFO
G
NID
DFO
H&E Staining
100μm
Ki67 Staining
100 μm
H
Ki67+ cell (%)
**
NID
DFO
I
NID
DFO
CRC Organoids
500 μm
J
Diameter (μm)
**
NID
DFO

Figure S2

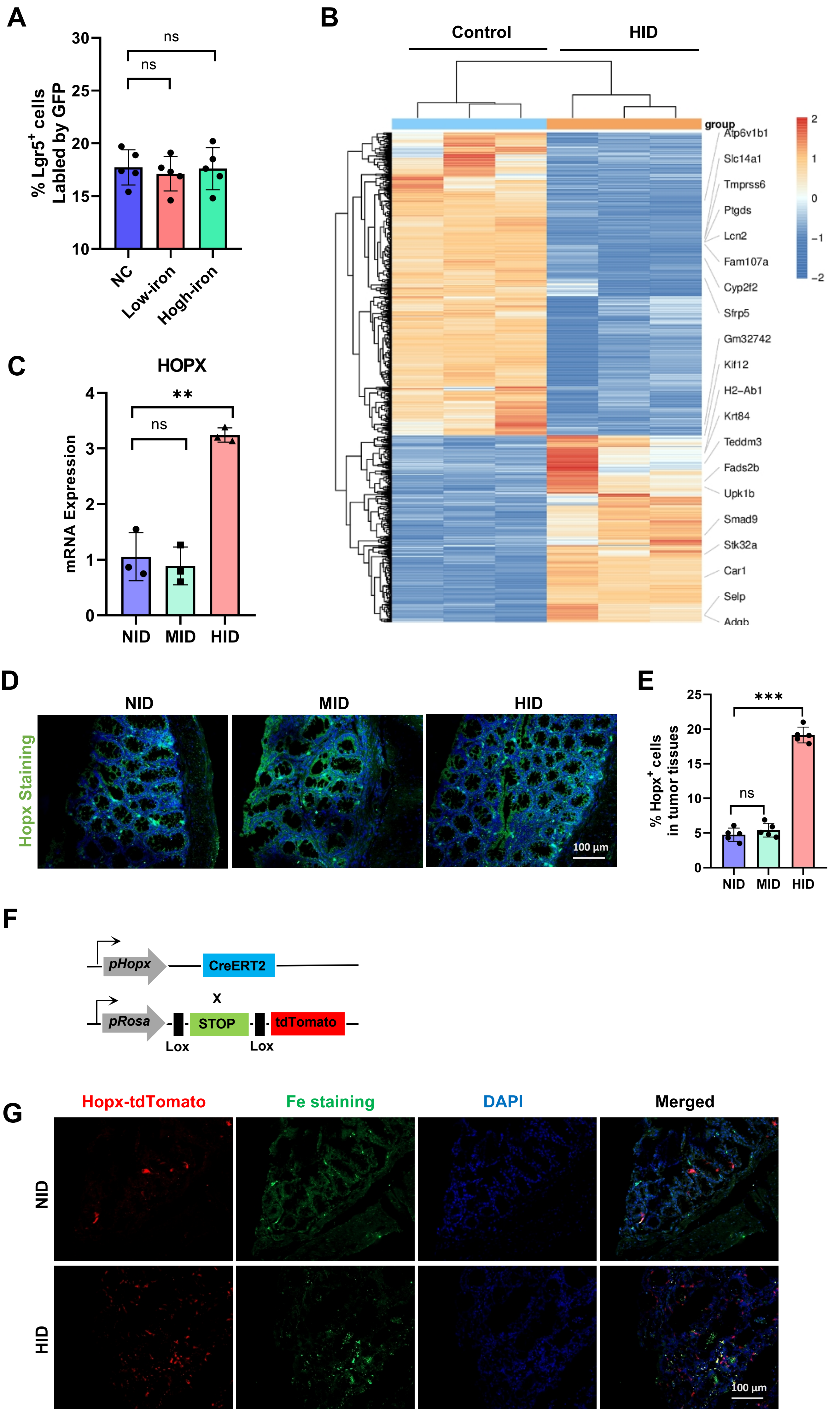
A
% Lgr5+ cells Labled by GFP
30
25
20
15
10
ns
ns
NC
Low-iron
Hogh-iron
B
Control
HID
group
2
1
0
−1
−2
Atp6v1b1
Slc14a1
Tmprss6
Ptgds
Lcn2
Fam107a
Cyp2f2
Sfrp5
Gm32742
Kif12
H2−Ab1
Krt84
Teddm3
Fads2b
Upk1b
Smad9
Stk32a
Car1
Selp
Adqb
C
HOPX
mRNA Expression
4
3
2
1
0
**
ns
NID
MID
HID
D
NID
MID
HID
Hopx Staining
100 µm
E
% Hopx+ cells in tumor tissues
25
20
15
10
5
0
***
ns
NID
MID
HID
F
pHopx
CreERT2
X
pRosa
STOP
tdTomato
Lox
Lox
G
Hopx-tdTomato
Fe staining
DAPI
Merged
NID
HID
100 µm

Figure S3

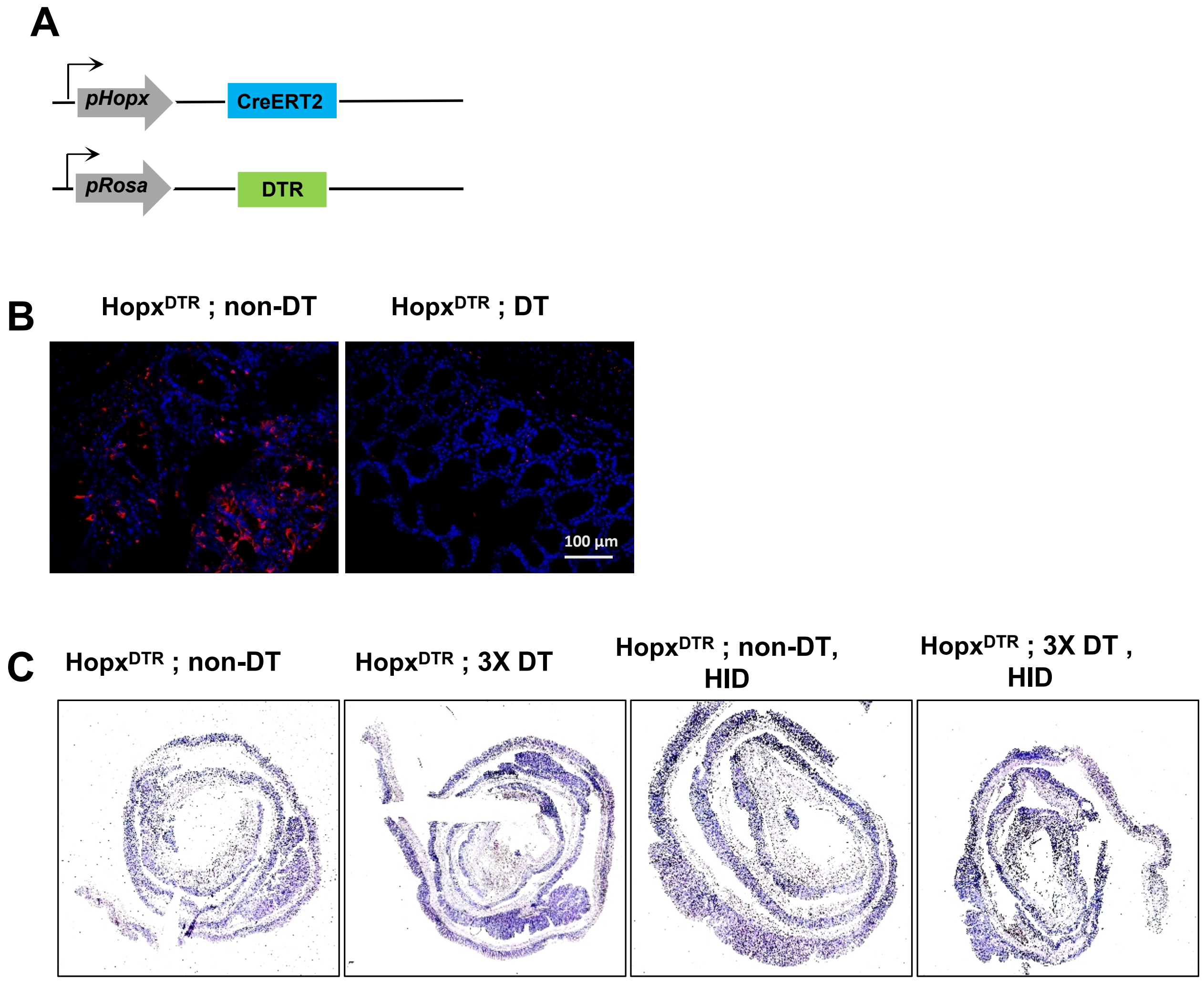
A
pHopx
CreERT2
pRosa
DTR
B
Hopx^{DTR} ; non-DT
Hopx^{DTR} ; DT
100 µm
C
Hopx^{DTR} ; non-DT
Hopx^{DTR} ; 3X DT
Hopx^{DTR} ; non-DT, HID
Hopx^{DTR} ; 3X DT , HID

Figure S4

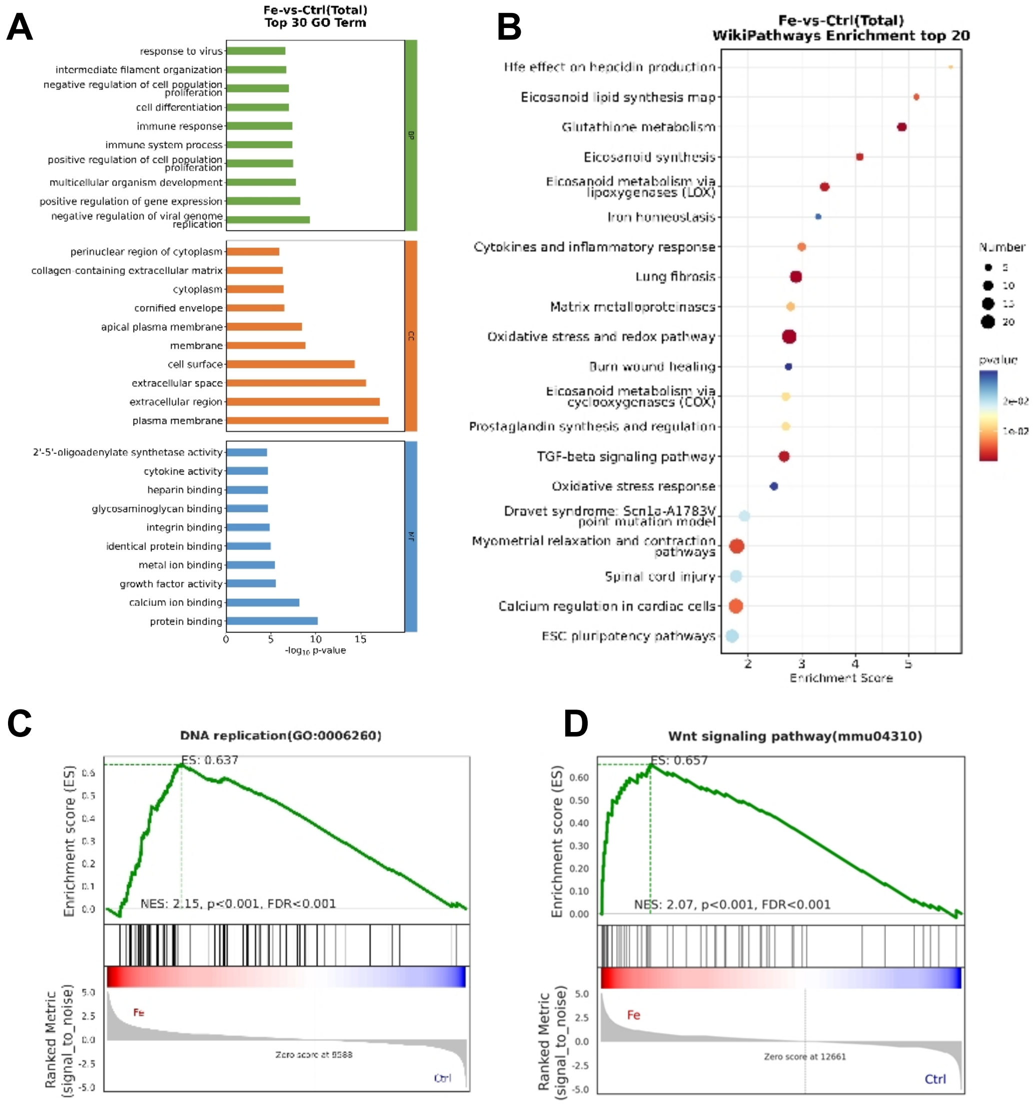
A
Fe-vs-Ctrl(Total)
Top 30 GO Term
response to virus
intermediate filament organization
negative regulation of cell population proliferation
cell differentiation
immune response
immune system process
positive regulation of cell population proliferation
multicellular organism development
positive regulation of gene expression
negative regulation of viral genome replication
perinuclear region of cytoplasm
collagen-containing extracellular matrix
cytoplasm
cornified envelope
apical plasma membrane
membrane
cell surface
extracellular space
extracellular region
plasma membrane
2'-5'-oligoadenylate synthetase activity
cytokine activity
heparin binding
glycosaminoglycan binding
integrin binding
identical protein binding
metal ion binding
growth factor activity
calcium ion binding
protein binding
-log10 p-value
B
Fe-vs-Ctrl(Total)
WikiPathways Enrichment top 20
Hfe effect on hepcidin production
Eicosanoid lipid synthesis map
Glutathione metabolism
Eicosanoid synthesis
Eicosanoid metabolism via lipoxygenases (LOX)
Iron homeostasis
Cytokines and inflammatory response
Lung fibrosis
Matrix metalloproteinases
Oxidative stress and redox pathway
Burn wound healing
Eicosanoid metabolism via cyclooxygenases (COX)
Prostaglandin synthesis and regulation
TGF-beta signaling pathway
Oxidative stress response
Dravet syndrome: Scn1a-A1783V point mutation model
Myometrial relaxation and contraction pathways
Spinal cord injury
Calcium regulation in cardiac cells
ESC pluripotency pathways
Enrichment Score
Number
pvalue
C
DNA replication(GO:0006260)
ES: 0.637
NES: 2.15, p<0.001, FDR<0.001
Enrichment score (ES)
Ranked Metric (signal_to_noise)
Fe
Ctrl
D
Wnt signaling pathway(mmu04310)
ES: 0.657
NES: 2.07, p<0.001, FDR<0.001
Zero score at 12661
Enrichment score (ES)
Ranked Metric (signal_to_noise)
Fe
Ctrl

Figure S5

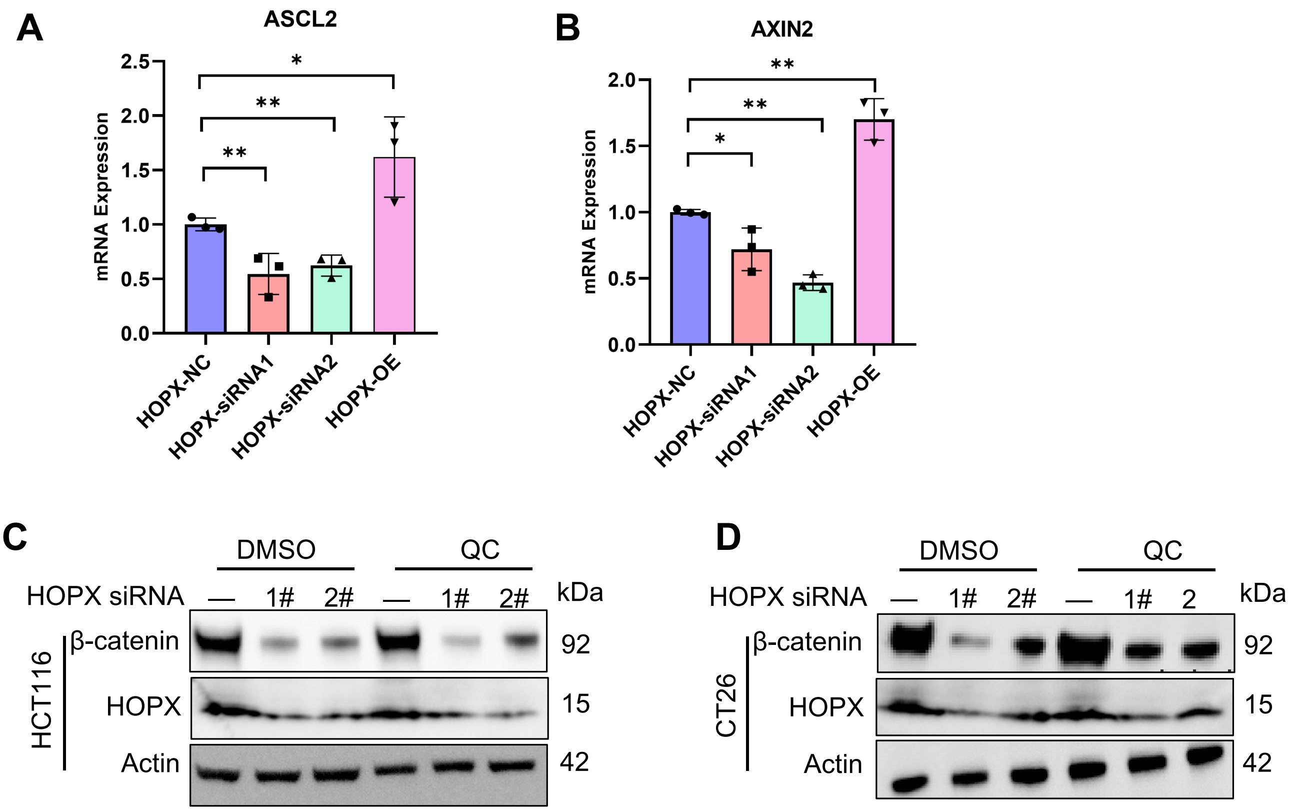
A
ASCL2
mRNA Expression
2.5
2.0
1.5
1.0
0.5
0.0
*
**
**
HOPX-NC
HOPX-siRNA1
HOPX-siRNA2
HOPX-OE
B
AXIN2
mRNA Expression
2.0
1.5
1.0
0.5
0.0
**
**
*
HOPX-NC
HOPX-siRNA1
HOPX-siRNA2
HOPX-OE
C
DMSO
QC
HOPX siRNA
—
1#
2#
—
1#
2#
kDa
HCT116
β-catenin
92
HOPX
15
Actin
42
D
DMSO
QC
HOPX siRNA
—
1#
2#
—
1#
2
kDa
CT26
β-catenin
92
HOPX
15
Actin
42

Figure S6

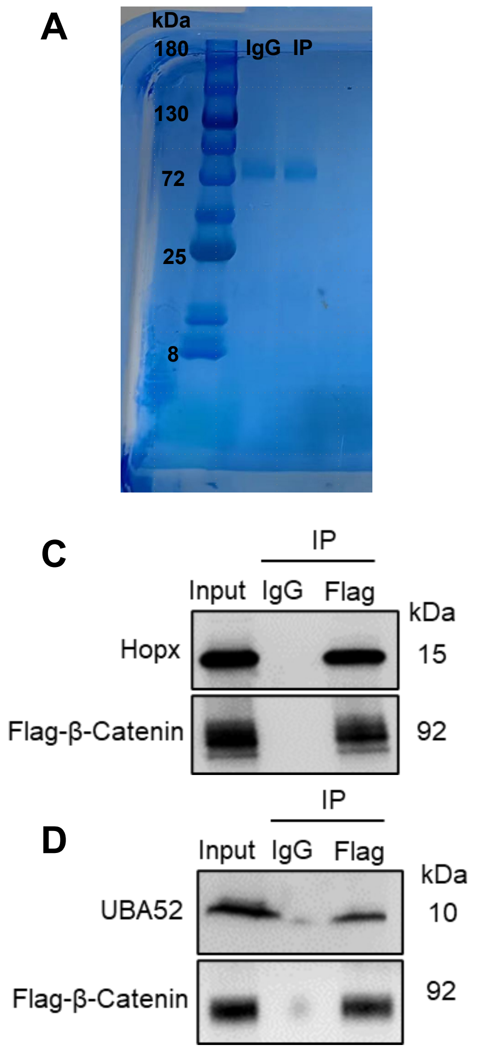

B

Gene name	Protein score	Sequence coverage (%)	Peptides	Abundances
Actg1	542	40	13	7.37e+07
Hsp90aa1	387	19	8	3.29e+07
Tuba1b	384	28	9	2.41e+07
H2bc21	379	35	1	2.60e+06
H2bc3	375	36	2	5.86e+07
H4c1	367	56	7	7.66e+07
Eef1a1	336	21	8	4.53e+07
Hsp90ab1	262	12	3	1.07e+07
H2ac20	209	36	4	5.07e+07
Hnrnpa1	183	11	2	
Hbb-b1	181	33	4	2.64e+07
H1-2	177	18	4	6.86e+06
Alb	176	7	4	7.20e+06
Uba52	167	31	4	1.44e+07
Hba	165	33	4	2.19e+07
Atp5f1b	162	14	5	1.25e+06
Tubb5	161	15	5	8.23e+06
Hspa1a	156	11	3	2.50e+06
Hspa8	111	5	1	

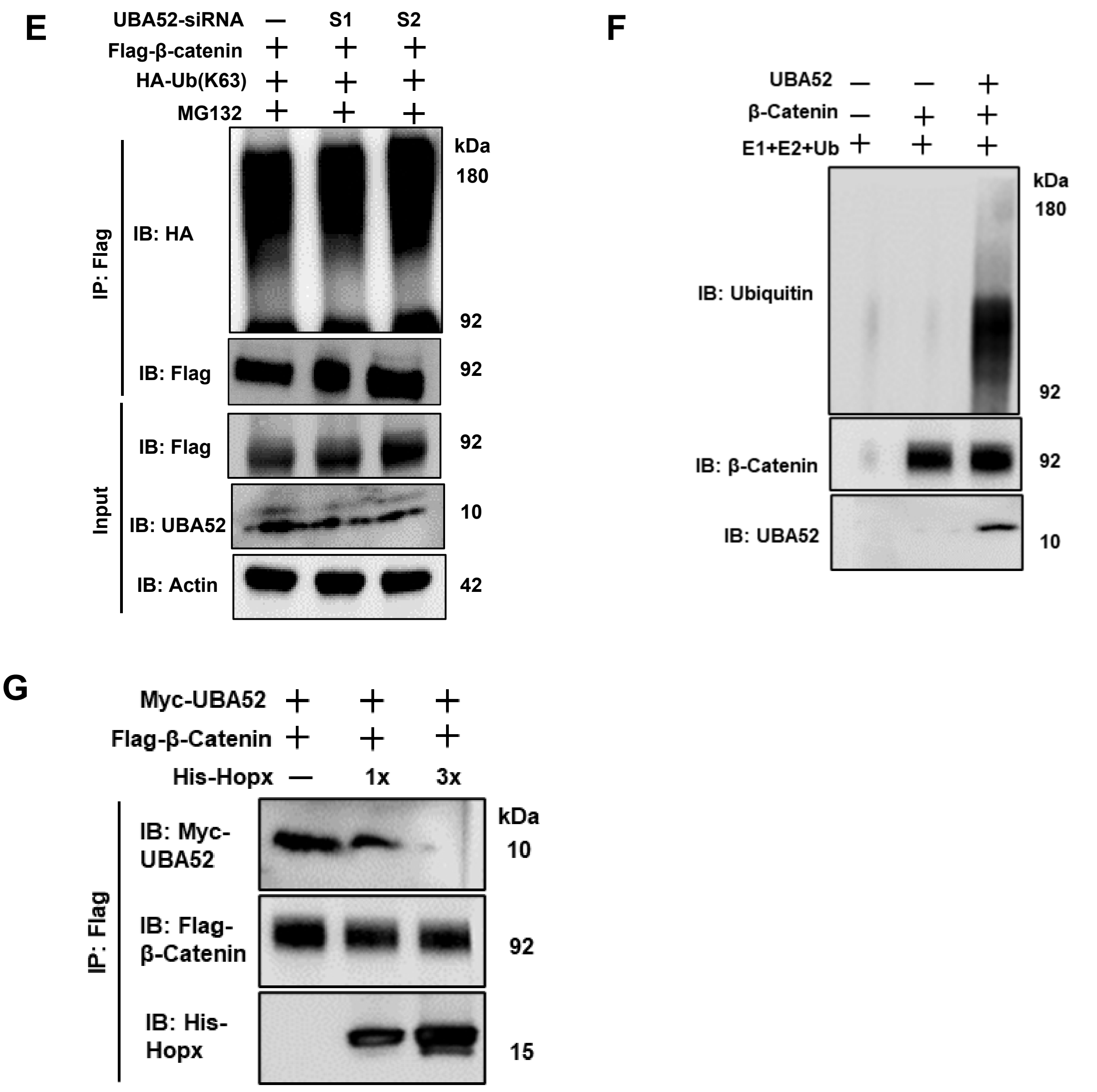

Figure S7

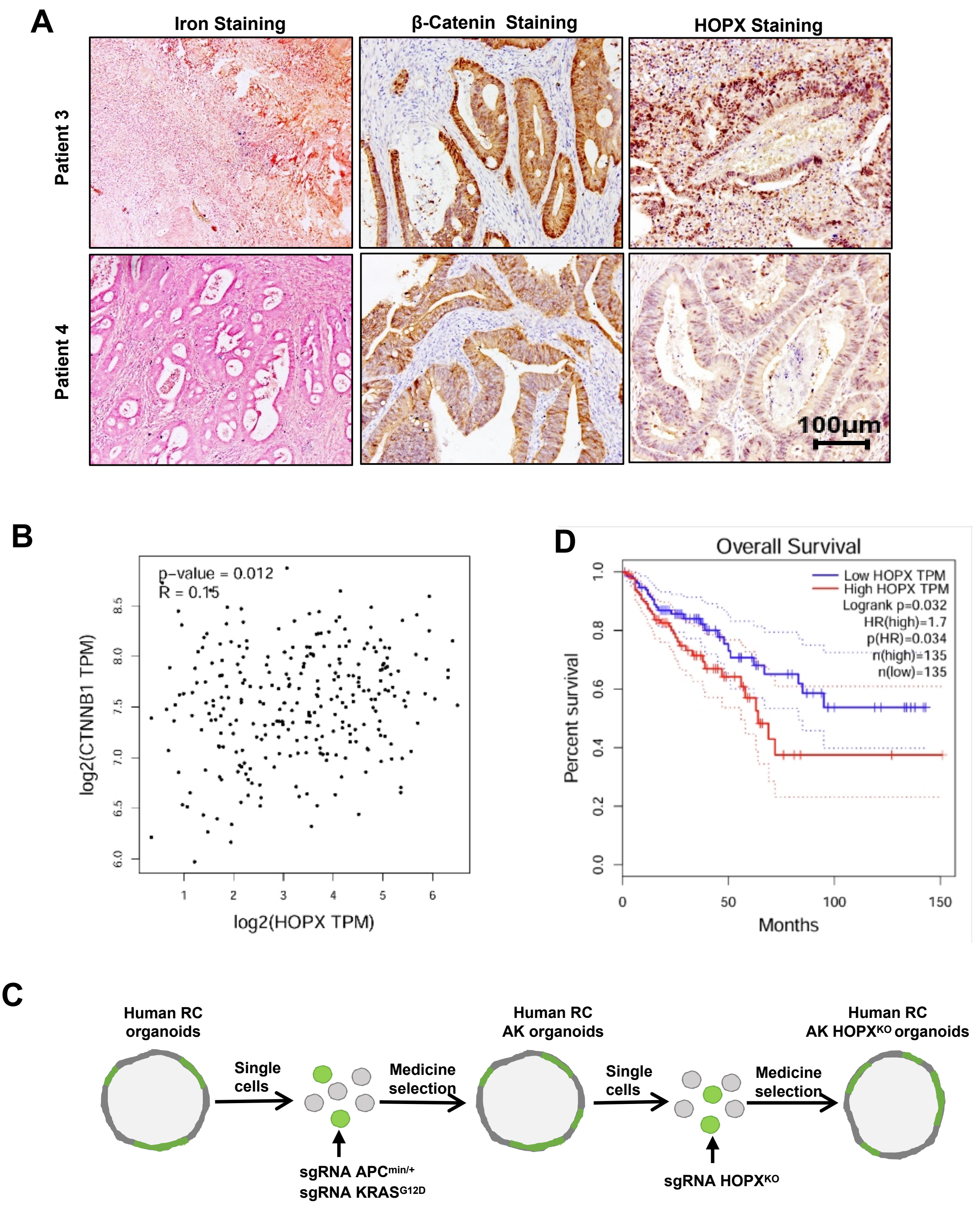
A
Iron Staining
β-Catenin Staining
HOPX Staining
Patient 3
Patient 4
100μm
B
p-value = 0.012
R = 0.15
log2(CTNNB1 TPM)
log2(HOPX TPM)
D
Overall Survival
Low HOPX TPM
High HOPX TPM
Logrank p=0.032
HR(high)=1.7
p(HR)=0.034
n(high)=135
n(low)=135
Percent survival
Months
C
Human RC organoids
Single cells
sgRNA APCmin/+
sgRNA KRASG12D
Medicine selection
Human RC AK organoids
Single cells
sgRNA HOPXKO
Medicine selection
Human RC AK HOPXKO organoids

Supplementary Tables

Supplementary Table 1. Quantitative RT-PCR primer

Target	Forward primer	Reverse primer
Hopx	TCAACAAGGTCGACAAGCAC	GTGACGGATCTGCACTCTGA
Axin2	TGACTCTCCTTCCAGATCCCA	TGCCCACACTAGGCTGACA
ASCL2	AAGCACACCTTGACTGGTACG	AAGTGGACGTTTGCACCTTCA
Ctnnb1	ATGGAGCCGGACAGAAAAGC	CTTGCCACTCAGGGAAGGA
b-actin	CTTCTTTGCAGCTCCTTCGTT	TTCTGACCCATTCCCACCA

Supplementary Table 2. siRNA sequences

Hopx	Forward primer	Reverse primer
S1	GCAGAUCUGUUACGGACUATT	UAGUCCGUAACAGAUCUGCTT
S2	GGCUUCUAUUGAAAAGAUATT	UAUCUUUUCAAUAGAAGCCTT
UBA52	Forward primer	Reverse primer
S1	GUCAGCUUGCCCAGAAGUACA	UGUACUUCUGGGCAAGCUGAC
S2	UCCAUCCACUGGAGCAGUAAA	UUUACUGCUCCAGUGGAUGGA

Supplementary Table 3. Mass spectrometry analysis results for the anti-His (His-tagged Hopx) immunoprecipitation complex.

Gene name	MW [kDa]	Protein score	Sequence coverage (%)	Unique Peptides	Peptides
Actg1	41.8	542	40	13	13
Hsp90aa1	84.7	387	19	8	11
Tuba1b	50.1	384	28	9	9
H2bc21	14	379	35	1	4
H2bc3	13.9	375	36	2	5
H4c1	11.4	367	56	7	7
Eef1a1	50.1	336	21	8	8
Hsp90ab1	83.2	262	12	3	6
H2ac20	14	209	36	4	4
Hnrnpa1	34.2	183	11	2	2
Hbb-b1	15.8	181	33	4	4
H1-2	21.3	177	18	4	4
Alb	68.6	176	7	4	4
Uba52	14.7	167	31	4	4
Hba	15.1	165	33	4	4
Atp5f1b	56.3	162	14	5	5
Tubb5	49.6	161	15	5	5
Hspa1a	70	156	11	3	5
Hspa8	70.8	111	5	1	3

Ldha	36.5	106	8	1	3
Atp5f1a	59.7	102	5	2	2
Ldhb	36.5	102	8	1	3
Jup	81.7	98	4	3	3
Rps3	26.7	84	10	2	2
Eif4a1	46.1	82	7	3	3
Ppia	18	82	16	2	2
Rps25	13.7	71	15	2	2
Ighg1	43.4	70	12	2	2
Eno3	47	65	3	1	1
H3-3a	15.3	63	13	2	2
Hnrnpc	34.4	59	4	1	1
Prdx1	22.2	52	15	2	2
C1qc	26	51	8	2	2
Trap1	80.2	51	2	1	1
Rpl18a	20.7	50	7	1	1
Gapdh	35.8	50	5	2	2
Lyz1	16.8	49	8	1	1
Prdx2	21.8	48	6	1	1
Ptma	12.2	47	13	1	1
Nucb2	50.3	46	2	1	1
Ywhaz	27.8	46	6	1	1
Eef2	95.3	45	2	2	2
Slc25a31	35.2	45	4	1	1
Tpi1	26.7	44	6	1	1
Mup17	20.6	43	6	1	1
Eef1g	50	43	3	1	1
Pde8b	96.7	39	1	1	1
Dsp	332.7	39	0	1	1
Hspd1	60.9	38	2	1	1
Eno1	47.1	37	3	1	1
Rps15a	14.8	37	6	1	1
Rps4x	29.6	37	3	1	1
Lonp1	105.8	36	1	1	1
Rpl8	28	35	4	1	1
Fam83e	52.5	34	1	1	1
Adgrv1	687	33	0	1	1
Rpl12	17.8	32	5	1	1
Dsg1a	114.5	31	1	1	1
Rps16	16.4	31	7	1	1
Rpl13	24.3	22	5	1	1
